# The novel and taxonomically restricted *Ah24* gene from grain amaranth (*Amaranthus hypochondriacus*) has a dual role in development and defense

**DOI:** 10.3389/fpls.2015.00602

**Published:** 2015-08-05

**Authors:** Julio A. Massange-Sanchez, Paola A. Palmeros-Suarez, Norma A. Martinez-Gallardo, Paula A. Castrillon-Arbelaez, Hamlet Avilés-Arnaut, Fulgencio Alatorre-Cobos, Axel Tiessen, John P. Délano-Frier

**Affiliations:** ^1^Biotechnology and Biochemistry Department, Centro de Investigación y de Estudios Avanzados del I. P. N., Unidad IrapuatoIrapuato, México; ^2^Facultad de Ciencias Biológicas, Instituto de Biotecnología, Universidad Autónoma de Nuevo LeónSan Nicolás de los Garza, México; ^3^The Sainsbury Laboratory, University of CambridgeCambridge, UK

**Keywords:** (a)biotic stress, defoliation, development, grain amaranth, jasmonic acid, taxonomically restricted genes

## Abstract

Grain amaranths tolerate stress and produce highly nutritious seeds. We have identified several (a)biotic stress-responsive genes of unknown function in *Amaranthus hypochondriacus*, including the so-called *Ah24* gene. *Ah24* was expressed in young or developing tissues; it was also strongly induced by mechanical damage, insect herbivory and methyl jasmonate and in meristems and newly emerging leaves of severely defoliated plants. Interestingly, an *in silico* analysis of its 1304 bp promoter region showed a predominance of regulatory boxes involved in development, but not in defense. The *Ah24* cDNA encodes a predicted cytosolic protein of 164 amino acids, the localization of which was confirmed by confocal microscopy. Additional *in silico* analysis identified several other Ah24 homologs, present almost exclusively in plants belonging to the Caryophyllales. The possible function of this gene *in planta* was examined in transgenic *Ah24* overexpressing *Arabidopsis thaliana* and *Nicotiana tabacum* plants. Transformed Arabidopsis showed enhanced vegetative growth and increased leaf number with no penalty in one fitness component, such as seed yield, in experimental conditions. Transgenic tobacco plants, which grew and reproduced normally, had increased insect herbivory resistance. Modified vegetative growth in transgenic Arabidopsis coincided with significant changes in the expression of genes controlling phytohormone synthesis or signaling, whereas increased resistance to insect herbivory in transgenic tobacco coincided with higher jasmonic acid and proteinase inhibitor activity levels, plus the accumulation of nicotine and several other putative defense-related metabolites. It is proposed that the primary role of the *Ah24* gene in *A. hypochondriacus* is to contribute to a rapid recovery post-wounding or defoliation, although its participation in defense against insect herbivory is also plausible.

## Introduction

Grain amaranths are C4 dicotyledonous plants that belong to the Amaranhaceae family and the Caryophyllales order, which groups several plant species with interesting physiological and agronomical characteristics (Stallknecht and Schulz-Schaeffer, 1993; Brenner et al., 2000). Grain amaranths, namely *Amaranthus hypochondriacus, A. cruentus*, and *A. caudatus* represent part of the approximately 60 *Amaranthus* species, that also include pernicious weeds or plants used as vegetables, ornamentals or fodder (Brenner et al., 2000; Venskutonis and Kraujalis, 2013; Giacomini et al., 2014). Grain amaranths are known to be tolerant to adverse environmental conditions, including drought, poor and/or saline soils and intense illumination (Brenner et al., 2000). Some of the physiological properties that have been associated with grain amaranth's positive agronomical traits are their C4 photosynthetic habit (Miller et al., 1984), high water use efficiency (Johnson and Henderson, 2002; Omami et al., 2006), an indeterminate flowering habit, and the ability to develop long tap-roots together with an extensive web of lateral roots (Miller et al., 1984; Li et al., 1989; Johnson and Henderson, 2002; Grobelnik-Mlakar et al., 2012). Other determinants of stress resistance have also been identified, including the ability to accumulate compatible solutes (Huerta-Ocampo et al., 2011), and the expression of stress-related genes (Huerta-Ocampo et al., 2009, 2014; Aguilar-Hernández et al., 2011) and transcription factors (Huerta-Ocampo et al., 2011). Additionally, grain amaranths show exceptional tolerance to severe defoliation (Castrillón-Arbeláez et al., 2012; Vargas-Ortiz et al., 2013), which was found to be associated with an efficient utilization of relatively high levels of stored carbon reserves in stem and roots. They are also known to readily respond to chemical elicitors of defense responses, such as jasmonic acid (JA) (Délano-Frier et al., 2004, 2011; Sánchez-Hernández et al., 2004) or benzothiadiazole (Casarrubias-Castillo et al., 2014), to increase their resistance against highly damaging sucking insect pests, such as the tarnished bug *Lygus lineolaris*, or against potentially lethal pathogenic bacteria.

The objective of this study was to characterize the novel stress-inducible *Ah24* gene, of unknown function, that was isolated from leaves of grain amaranth plants. The gene's nomenclature originated from its position (clone number 24) in a micro-plate that contained part of the SSH library obtained from leaves of *A. hypochondriacus* plants subjected to various stress treatments, including insect herbivory (Délano-Frier et al., 2011). This gene was subsequently found to be expressed at high levels in response to mechanical wounding, insect herbivory and JA (Navarro-Meléndez, 2009; Massange-Sánchez, 2011). Additionally, a recent proteomic study found that Ah24 accumulated in *A. cruentus* roots in response to salt stress (Huerta-Ocampo et al., 2014). This pattern indicated that it could be a novel and important regulator of (a)biotic stress responses in grain amaranth and perhaps other related species (Navarro-Meléndez, 2009; Fomsgaard et al., 2010; Délano-Frier et al., 2011; Massange-Sánchez, unpublished data). This was supported by subsequent searches indicating that Ah24 and homologous proteins are taxonomically restricted, since they are almost exclusively found in plants belonging to the Caryophyllales order, which includes many extremophytes (Oh et al., 2012). On the basis of these compelling results, a further study was undertaken to perform an ample characterization of this gene in order to gain a deeper insight into its function *in planta*. With this objective in mind, the complete gene and cDNA sequences of the *Ah24* gene were obtained, together with a sizeable section of its promoter region, which was found to have elements related not only to defense-related JA responses, but also to development. Gene expression analysis in *A*. *hypochondriacus* showed that this gene is expressed in actively growing tissues and confirmed its strong response to methyl jasmonate (MeJA) and insect herbivory. Moreover, a functional characterization of this gene using a transgenic approach in *Arabidopsis thaliana* and *Nicotiana tabacum* confirmed its positive role not only in defense against herbivory, but also in vegetative growth regulation. The above traits might contribute to the high tolerance to defoliation (Castrillón-Arbeláez et al., 2012; Vargas-Ortiz et al., 2013) and chewing insect herbivory (Brenner et al., 2000; Niveyro and Salvo, 2014) previously reported in grain amaranth. Moreover, the overall results indicate that taxonomically restricted genes, mostly found in plant orders rich in extremophytes, such as the Caryophyllales, can be utilized to engineer stress resistance in commercially important crops, such as rice, corn and wheat, which are less tolerant to adverse environmental conditions.

## Results

### Isolation of the *Amaranthus hypochondriacus Ah24* gene and coding region

**Figure 1:**
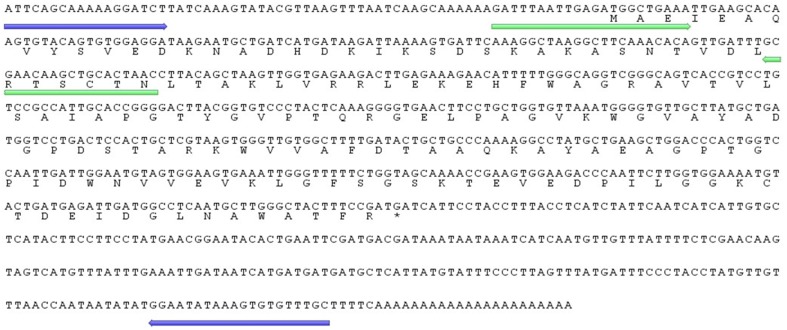
**Complete 847 bp cDNA sequence of the ***Ah24*** gene and the predicted 164 amino acid protein coded by its open reading frame**. Shown in blue and green are sequence regions selected to design the primers used to isolate its genomic sequence by a gene walking strategy and to quantify the *Ah24* expression levels by RT-qPCR, respectively.

Employing PCR-based cDNA subtraction, a partial *Ah24* cDNA clone of 858 bp, containing part of the 5′ UTR region, was obtained from combined cDNA libraries of grain amaranth plants subjected to water- and salt-stress, insect herbivory, and mechanical damage (Fomsgaard et al., 2010). Using 5′ RACE PCR, a full-length 844 bp *Ah24* cDNA clone was obtained, including a 65 bp 5′UTR, 492 bp open reading frame (ORF), and 287 bp 3′UTR (GenBank accession number JN384107; Figure 1). The ORF encodes a polypeptide of 164 amino acid residues with a predicted molecular mass of 17.6 kDa and pI of 5.71 (Figure 1). The prediction was corroborated by an 82% identity with fingerprint peptides derived from a proteomic analysis of an *A. hypochondriacus* protein induced by exogenous MeJA (Navarro-Meléndez, 2009; Figure S1 in Supplementary Materials). Bioinformatic analyses further indicated that the secondary structure of the Ah24 protein includes 6 α helices and 3 β-sheets (Figure S2A in Supplementary Materials). Also predicted were one possible N glycosylation site (N_41_L_42_) and four putative protein kinase C tyrosine phosphorylation sites, at positions Y_43_, Y_74_, Y_98_, and Y_162_ (Figure S2B in Supplementary Materials).

**Figure 2:**
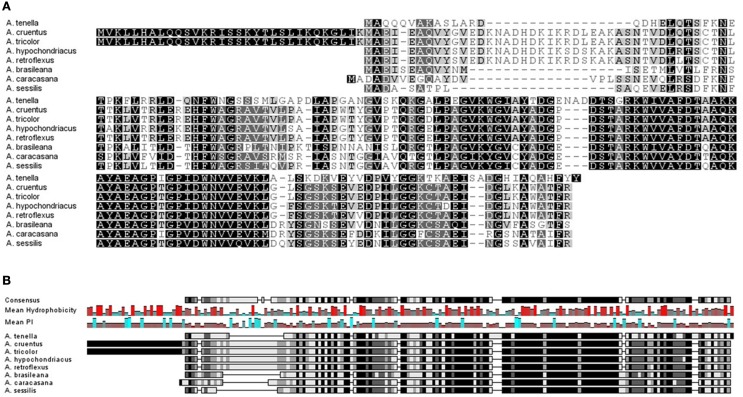
**In (A), the amino-acid alignment of the Ah24 protein from ***Amaranthus hypochondriacus*** with closely related proteins identified in other ***Amaranthus*** species and in ***Alternanthera*** spp. is shown**. Ah24 and related *Amaranthus* proteins share the highest degree of homology with proteins identified in various species of the latter genus (i.e., *A. brasiliana, A*. *caracasana, A*. *sessilis*, and *A*. *tenella*). In **(B)**, the predicted consensus level, mean hydrophobicity, and mean isoelectric point of Ah24 and closely related proteins is shown.

**Figure 3:**
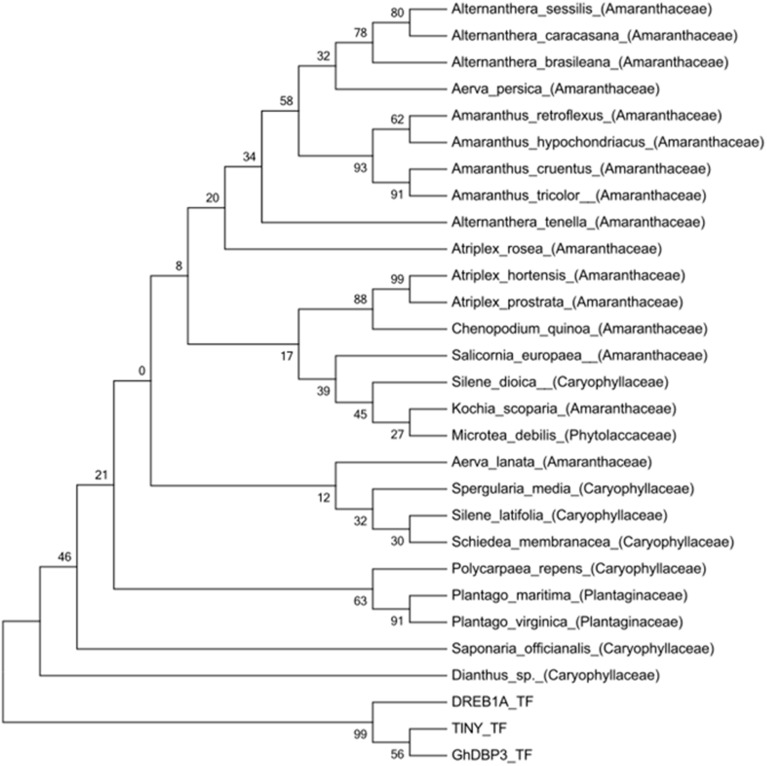
**Phylogenetic tree of the Ah24 protein of ***A. hypochondriacus*** and homologous proteins in related plant species**. The phylogenetic tree was constructed by the neighbor-joining method with amino-acid sequence data obtained from the1000 Plants initiative as described in the text. It was drawn using TreeView based on alignments obtained using MUSCLE software. The bootstrap values shown are in percent. DREB1a, TINY and GhDBP3 ERF TFs are included as an external group.

An initial search in the NCBI and EMBL databases for nucleotide and protein sequences retrieved two hits for the *Ah24* cDNA or protein, both corresponding to other *Amaranthus* species (see below). However, an *in silico* comparison with the predicted proteins codified by the transcriptomic data generated by the 1000 Plants Initiative (refer to Materials and Methods for hyperlink) which includes medicinal plants and extremophytes, showed that the Ah24 protein shared closest homology to other predicted Ah24 proteins in *Amaranthus* and *Alternanthera* (Figure 2) and, to a lesser degree, to predicted proteins in plants belonging almost exclusively to the Caryophyllales order (Figure 3). Not surprisingly, most of the *Alternanthera* species in which Ah24 homologs have been identified are considered noxious weeds (Tanveer et al., 2013).

A *gene walking* experimental strategy permitted the isolation of the *Ah24* gene, consisting of 1881 bp organized into two introns (701 and 296 bp in length, respectively) and three exons (37, 365, and 442 bp in length, respectively). Furthermore, gene-specific primers amplified 1304 bp of the promoter region of this gene (GenBank accession number KP184919; Figure S3 in Supplementary Materials). Cis-acting regulatory element analysis (Genomatix 8.0.5) identified 42 regulatory motifs in the promoter region (Table S1 in Supplementary Materials), of which the AHBP, CCAF, GTBX, L1BX, and MADS motifs, mostly involved in the control of developmental processes and/or stress responses, were particularly abundant, comprising more than 50% of the motifs identified in the *Ah24* gene promoter region analyzed (Table S2 and Figure S4 in Supplementary Materials). G-boxes and unusual TGACG motifs, both associated with the response to JA (Memelink, 2009), were also detected (Table S2 in Supplementary Materials).

### Tissue-specific pattern of *Ah24* expression and in response to (a)biotic stresses

**Figure 4:**
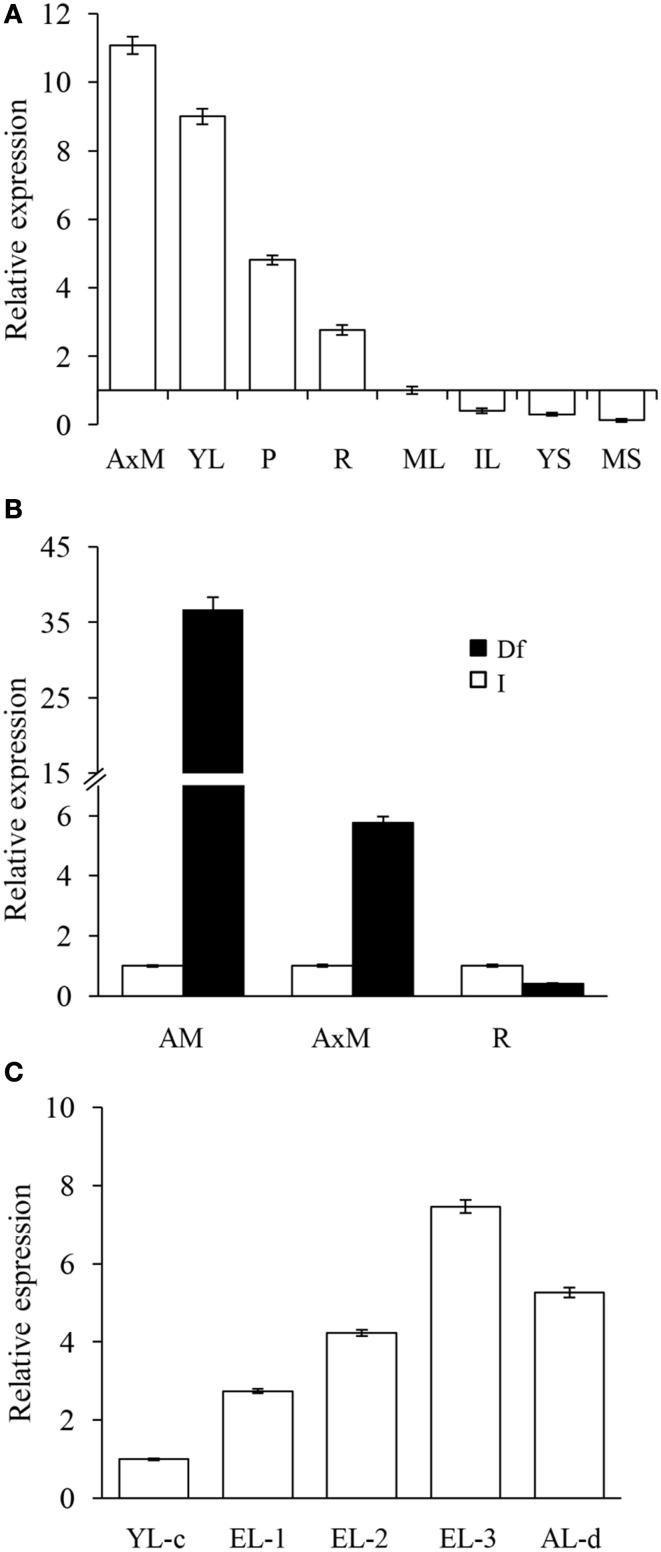
**The ***Ah24*** gene is expressed in rapidly developing tissues in intact and defoliated ***A. hypochondriacus*** plants**. **(A)** Shows that the *Ah24* gene is expressed in rapidly developing tissues, such as axilar meristems (AxM), young leaves (YL), panicles (P), and roots (R). The basal tissue-specific expression levels shown are relative to those detected in mature leaves (ML), whose expression was set at 1. Other tissues tested were young and mature stems (YS and MS, respectively) and intermediate leaves (IL). Solid bars show the strong expression of this gene in apical (AM) and axillar (AxM) meristems, but not in roots **(B)**, and in emerging leaves (EL-1,-2, and -3; **C**) sampled from grain amaranth plants recovering from severe defoliation. Expression in defoliated plants (Df) was compared to the expression levels in equivalent tissues from intact plants (I), which were normalized to a value of 1, and shown as empty bars in **(B)**. In **(C)**, expression levels in defoliated plants are reported relative to young leaves (YL-c) of approximately the same age sampled from intact controls. EL-1, -2, and -3 were sampled at 3, 6, and 9 days after defoliation. The expression levels in the youngest leaf remaining in the apex of the defoliated plant (AL-d), which was sampled immediately after defoliation was completed, were also determined.

Real time PCR was employed to determine the basal expression levels of *Ah24* in *A. hypochondriacus* tissues at different developmental stages. *Ah24* was expressed predominantly in emerging or rapidly developing or expanding tissues, such as apical meristems, young leaves, roots, and panicles (Figure 4A). Strong levels of expression were also detected in apical meristems, stems (Figure 4B) and emerging leaves of young *A. hypochondriacus* plants recovering from severe defoliation (Figure 4C). Additionally, *Ah24* expression was very strongly activated by mechanic wounding, insect herbivory and MeJA treatment (Table 1). The expression patterns and maximum expression levels varied in response to the treatments applied, being clearly stronger in MeJA-treated plants. The local induction was very rapid and intense, becoming evident between 1 and 3 h post treatment (hpt) and reaching highest levels of expression between 9 and 12 hpt [mechanically wounded (W) and MeJA treatments] or at 36 hpt [herbivory (H) treatments]. Systemic induction in distal leaves of W and MeJA-treated plants was weaker and tended to be slower, particularly in W plants. Conversely, the expression of the *Ah24* gene in H-treated plants was higher in distal, intact leaves than in damaged leaves, with both types of tissues reaching maximum levels of *Ah24* expression at 36 hpt. The expression patterns differed, both in intensity and kinetics of expression, from those produced by *AhAOC*, coding for allene oxide synthase, a JA-biosynthetic enzyme, and *AhKTI*, coding for a Kunitz trypsin inhibitor, which were used as markers of the wound response (Castrillón-Arbeláez et al., 2012) (Table 1). Induced *Ah24* expression in leaves of *A. hypochondriacus* was also relatively stable, remaining up-regulated for up to 36 hpt. No W- or MeJA-induced expression of *Ah24* was detected in roots and stems of *A. hypochondriacus* plants subjected to the above treatments (results not shown). Also, exposure to water- or salt-stress did not induce the expression of *Ah24* in young amaranth plants (results not shown).

**Table 1:** **The ***Ah24*** gene is induced in leaves of ***A. hypochondriacus*** plants subjected to insect herbivory (H), mechanical wounding (W), or methyl jasmonate (MeJA) treatments**.

**Relative expression^a^**									
**Hours post treatment (hpt)**			**1**	**3**	**6**	**9**	**12**	**24**	**36**
*Ah24*	H	L^b^	–	25	55	61	313	147	688
		S^b^	–	6	20	6	37	212	722
	W	L	42	354	180	1064	1787	194	196
		S	1	–	–	32	23	4	3
	MeJA	L	13	416	3782	3713	1091	2905	322
		S	3	3	12	33	85	125	77
*AhAOS*^c^	H	L	–	14	11	06	14	2	15
		S	–	1	1	1	2	1	3
	W	L	102	18	2	1	3	–	1
		S	–	–	–	1	2	–	1
	MeJA	L	24	33	13	9	4	3	2
		S	3	3	6	14	6	6	9
*AhKTI*^c^	H	L	–	–	–	–	1	1	2
		S	–	1	1	1	2	1	3
	W	L	1	1	–	2	2	1	2
		S	1	–	–	7	5	2	3
	MeJA	L	3	11	13	23	13	12	7
		S	5	7	17	63	27	19	43

a The fold change in the expression of the target genes was calculated using the 2^−ΔΔCt^ method according to Livak and Schmittgen (2001); controls were leaves from intact plants (for herbivory and mechanical wounding assays) or from plants treated with 0.01% Triton X-100 only (for MeJA assay).

b L, local response; S, systemic response.

c AhAOC, A. hypochondriacus Allene oxide cyclase; AhKIT, A. hypochondriacus Kunitz trypsin inhibitor.

### Subcellular localization of Ah24

**Figure 5:**
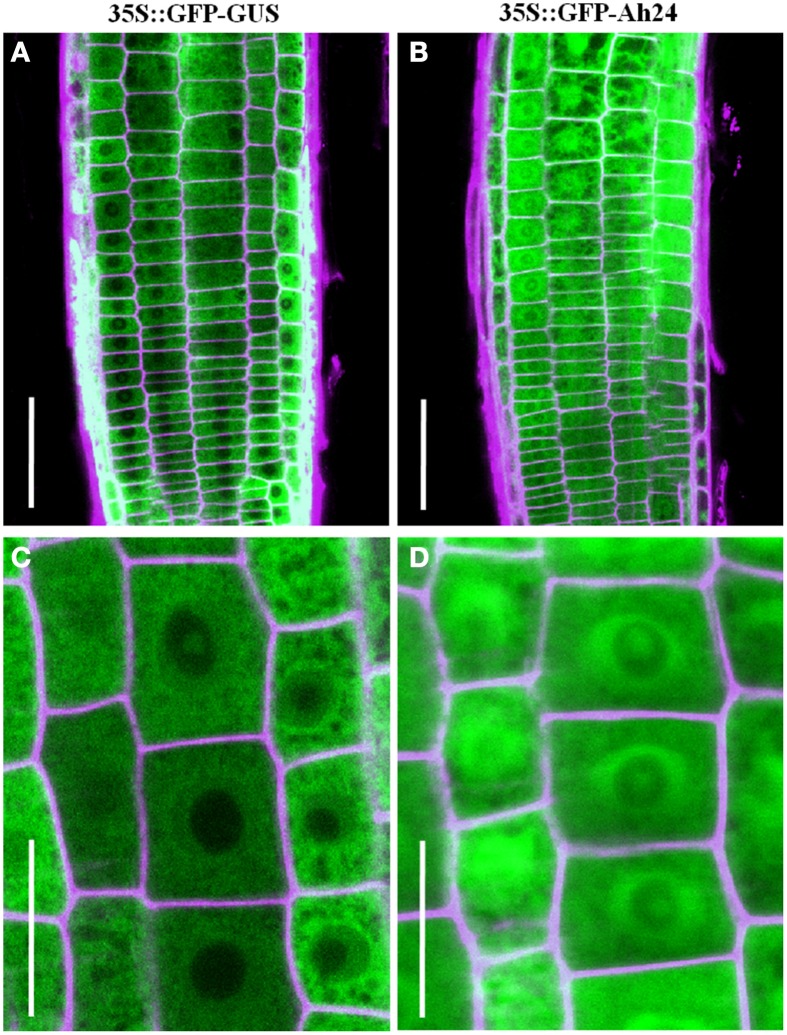
**Localization of the Ah24-GFP fusion protein by fluorescence microscopy in cells near the root tip**. A cytosolic **(B,D)** localization, in the periphery of the nucleus, and perhaps also in the nucleolus, was observed in root cells of transgenic Arabidopsis plants constitutively expressing the Ah24-GFP fusion protein. The results were compared with control transgenic Arabidopsis plants constitutively expressing a GF-GUS fusion protein **(A,C)**.

Prediction of subcellular localization using ProtComp v9.0 software suggested that Ah24 had a cytosolic localization. This prediction was supported by the expression and distribution of the Ah24-GFP chimeric protein in roots of transgenic *Ah24* OE Arabidopsis plants was tracked by fluorescence microscopy (Figure 5). Curiously, the marker protein showed a tendency to accumulate in the periphery of the nucleus. A possible localization in the nucleolus was also suggested by the fluorescence intensity.

### Generation and functional characterization of *Ah24* OE-transgenic *A. thaliana* and *N. tabacum* plants

**Figure 6:**
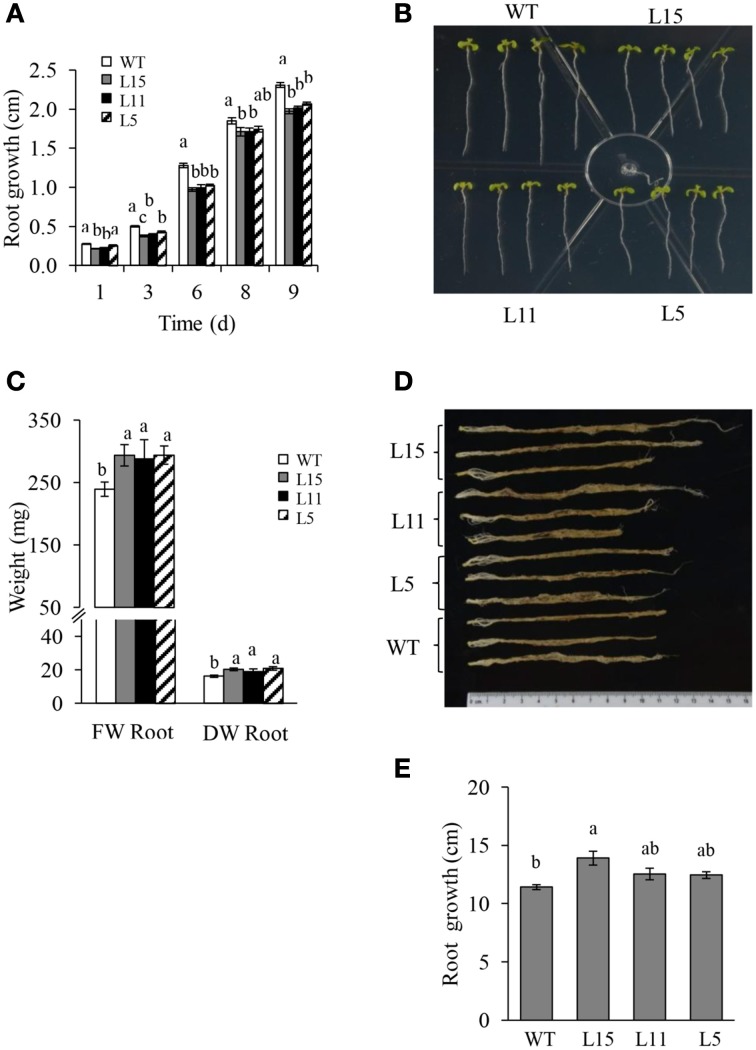
**The negative effect on root growth in young seedlings caused by the overexpression (OE) of ***Ah24*** in transgenic Arabidopsis plants is reversed as plant develops**. Bars and error bars represent the mean value ± SE of **(A)** primary root length of 2- to 9-days old seedlings grown vertically in Petri dishes, and of **(C)** root fresh (FW) and dry weights (DW) of soil-grown mature *Ah24* OE transgenic (L15, gray; L11, black, and L5, striped) and untransformed WT (empty bars) plants. **(B,D)** Shows images of the primary root length of 9-day-old seedlings, and of mature untransformed (WT) and transgenic *Ah24* OE plants (L15, L11 and L5). **(E)** Bars and error bars represent the mean root length ± SE of mature transgenic and WT plants shown in **(D)**. All data was obtained using six plants per analysis (*n* = 6). Different letters (a–c) over the bars represent statistically significant differences at *P* ≤ 0.05 (Tukey–Kramer test).

**Figure 7:**
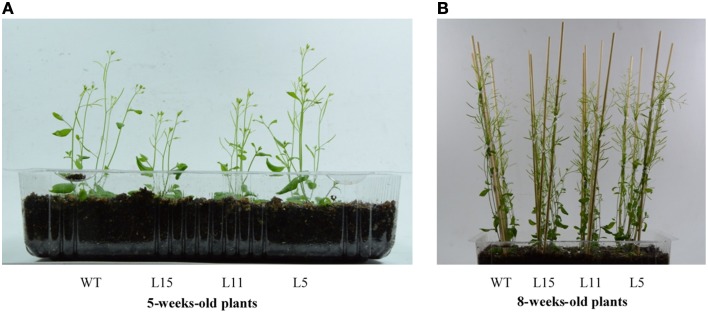
**The overexpression (OE) of ***Ah24*** in transgenic Arabidopsis plants temporarily affects the length of the main stem in a gene dosage-dependent manner**. In **(A)**, the length of the main stem in 5-weeks-old bolting plants grown in soil in the three *Ah24* OE transgenic lines and WT controls, is compared. In **(B)**, no differences in the length of the main stem between WT and *Ah24* OE transgenics is observed in 8-weeks-old, fully-developed plants grown in soil.

**Figure 8:**
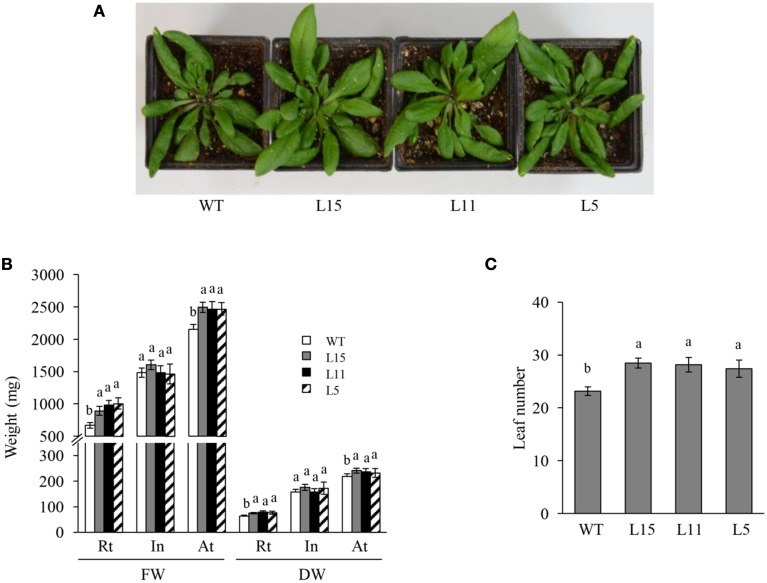
**The overexpression (OE) of ***Ah24*** in transgenic Arabidopsis plants enhances vegetative growth and increases leaf number**. **(A)** The rosette size of the three lines of transgenic Arabidopsis plants tested (L15, L11, and L5) is visibly larger than WT controls. Bars and error bars represent the mean values ± SE of **(B)** the fresh (FW) and dry weights (DW) of the rosette (Rt), inflorescence (In) and aerial tissue (At: Rt + In) of mature *Ah24* OE transgenic (L15, gray; L11, black, and L5, striped) and WT (empty bars) Arabidopsis plants, and of **(C)** the leaf number in mature transgenic *Ah24* OE (L15, L11, and L5) and untransformed (WT) plants. All data was obtained using 10 plants per analysis (*n* = 10). Different letters over the bars represent statistically significant differences at *P* ≤ 0.05 (Tukey–Kramer test).

Seven Arabidopsis T_2_ homozygous OE transgenic lines with a single T-DNA insertion were randomly selected. They showed significantly different *Ah24* expression levels, ranging from 1 × 10^4^ to 5 × 10^5^-fold higher expression levels relative to background expression levels in untransformed controls (Figure S5 in Supplementary Materials). Three lines with differing *Ah24* expression levels [high (L15), middle (L11), and low (L5)] were selected for further experimentation. In most experiments, *Ah24* expression dosage in transgenic OE Arabidopsis lines significantly affected their vegetative and reproductive growth. The primary roots of the three OE lines were significantly shorter than the WT controls as measured in 2- to 9-day-old seedlings (Figures 6A,B). However, this tendency was reversed as plants developed, since roots tended to be larger and had a higher fresh and dry weights in 6-week-old OE transgenic plants (Figures 6C–E). WT and OE plants bolted after 3 weeks of growth and developed a main stem. No difference in flowering time (i.e., 3.5 weeks ± 1 day) was detected between WT and OE plants. However, the length of the main stem was inversely correlated with the level of *Ah24* transgene expression, as shown in Figure 7A. Also, this negative effect was corrected as plants became older, since stem size in the L15 line became indistinguishable from WT plants (Figure 7B). Conversely, rosette sizes in OE plants were visibly larger, as confirmed by their significantly higher rosette and inflorescence fresh and dry masses (Figures 8A,B). Surprisingly, leaf number was significantly higher in all three transgenic OE lines tested (Figure 8C). These changes in vegetative development did not impose a fitness cost in terms of reproductive development since no differences in seed yield were observed between WT and transgenic OE plants (Figure S6 in Supplementary Materials).

Contrary to Arabidopsis, the OE of the *Ah24* gene in three of the three T_2_ lines of transgenic tobacco plants examined (Figure S7 in Supplementary Materials) had mostly a neutral effect on vegetative and reproductive growth, although the most highly expressing transgenic OE line (L14) showed a temporary slower growth rate, manifested at mid-development (Figure S8A in Supplementary Materials). Leaf number, seed capsule number and seed yield per plant in OE plants were not significantly different from those in WT (Figures S8B–D in Supplementary Materials). No difference in seed morphology and size was observed between seeds obtained from WT and transgenic Arabidopsis and tobacco plants.

**Figure 9:**
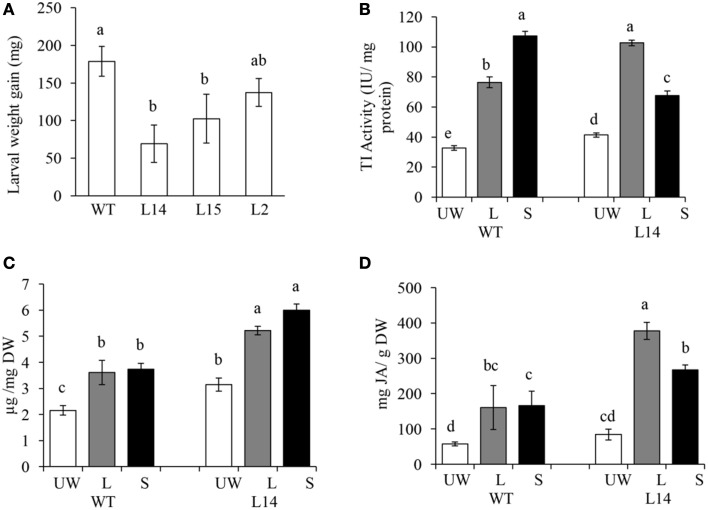
**The overexpression (OE) of ***Ah24*** in transgenic tobacco plants increases resistance to insect herbivory by leaf chewing larvae**. Bars and error bars represent the mean values ± SE of **(A)** the weight gain in *Manduca sexta* larvae which fed on *Ah24* OE transgenic plants having increasing transgene dosage [L2 (low), L15 (medium), and L14 (high)] or untransformed (WT) tobacco plants, and of **(B)** trypsin inhibitor activity **(C)** nicotine, and **(D)** jasmonic acid levels in unwounded leaves (UW; empty bars) and in damaged (local response, L; gray bars) and distal undamaged (systemic response, S; black bars) leaves of untransformed (WT) and transgenic *Ah24* OE (L14) tobacco plants. All data was obtained using six plants per analysis (*n* = 6). Different letters over the bars represent statistically significant differences at *P* ≤ 0.05 (Tukey–Kramer test).

**Figure 10:**
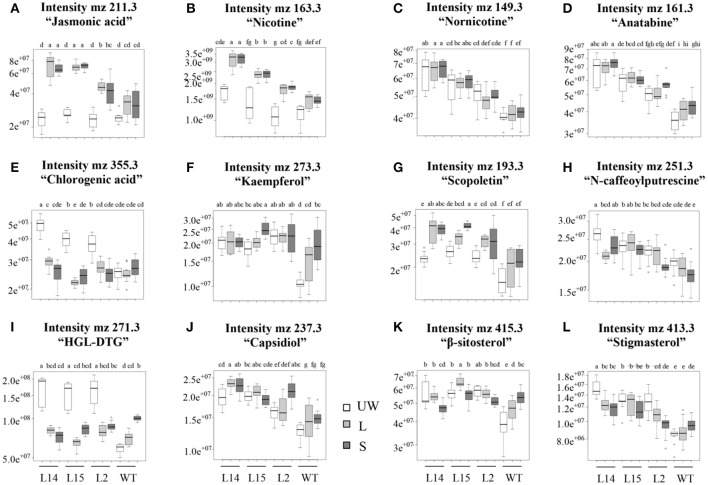
**Box-plots showing the levels (represented as different intensities of the respective m/z peaks) of 12 putative defense-related secondary metabolites in leaves of untransformed (WT) and of three ***Ah24*** overexpressing (OE) transgenic tobacco plants having differing transgene dosage (L14, L15, and L2)**. The bars represent the mean value ± SE (*n* = 120) of the peak intensities determined in unwounded leaves (UW, empty bars) and from wounded (local response, L; light gray bars) and intact (systemic response, S; dark gray bars) leaves of insect-damaged plants. Different letters over the bars represent statistically significant differences at *P* ≤ 0.05 (Tukey–Kramer test). Herbivory-induced increase of the m/z peaks representing putative jasmonic acid **(A)** and nicotine **(B)** levels coincided with the GC/MS data shown in Figure 9. m/z peaks that coincided with defense-related genes known to accumulate in tobacco in response to biotic stressors are shown in **(C–L)**.

Conversely, resistance to insect herbivory by *M. sexta* larvae in transgenic OE tobacco lines was clearly dependent on transgene dosage. Thus, only the highest *Ah24*-expressing L14 and L2 plants were able to significantly reduce larval body weight gain, as compared to WT controls, measured after 3-day continuous leaf feeding trials (Figure 9A). Resistance to insect herbivory in these *Ah24* transgenic tobacco lines correlated with constitutively high trypsin inhibitor levels in damaged leaves only (Figure 9B) and induced accumulation of nicotine (Figure 9C) and JA (Figure 9D). Significantly higher JA and nicotine levels correlated with data obtained from a parallel metabolomics analysis in which a similarly significant enrichment of ion peaks having an m/z-value that coincided with their respective molecular weights (Figures 10A,B). Twelve additional m/z ions that putatively correspond to secondary metabolites known to increase in response to biotic stress in tobacco (Figures 10C–L and Figure S9 in Supplementary Materials), plus eight other unidentified m/z ions, showed an altered pattern of accumulation in response to herbivory, as compared to WT plants (Figure S9 in Supplementary Materials).

### Microarray analysis of arabidopsis *Ah24* OE plants

To elucidate the effect that the OE of the *Ah24* transgene had in Arabidopsis gene expression, a microarray analysis of WT and L15 *Ah24* OE transgenic plants under normal growing conditions was performed. The fold change analysis (FC = 1.6) showed that the expression of 2602 genes was altered in the *Ah24* OE transgenic L15 line as compared with WT plants, of which 1293 were up-regulated and 1309 were down-regulated (Table S3 in Supplementary Materials). Of relevance was modified expression of various *MIR*-encoding genes whose targets impinge on plant hormone signaling (Schommer et al., 2008; Siré et al., 2009; Zhu et al., 2011; Barah et al., 2013; Turner et al., 2013; Curaba et al., 2014). Also represented were genes involved in the maintenance of gibberellin homeostasis (e.g., *CYP714A2* and *AGF1;* Zhu et al., 2006; Matsushita et al., 2007; Zhang et al., 2011) and in phytohormone signaling (e.g., *SLEEPY1*, coding for an F-box family protein gene that positively regulates gibberellin signaling; *CRF2*, for an ethylene-responsive transcription factor involved in cytokinin signal transduction, and *SEUSS-like 1*, for a transcriptional adaptor that regulates floral and embryonic development and also auxin-related phenotypic and transcriptional responses; Bao et al., 2010; Ariizumi et al., 2011; Cutcliffe et al., 2011). The *RHE1a* gene, coding for a RING finger E3 ligase, which is considered to be part of the overall signal involved in plant development and defense (Schwechheimer et al., 2009), was also found to be strongly down-regulated in *Ah24* OE Arabidopsis plants.

A gene ontology (GO) analysis of the microarray data was also performed (Table S3 in Supplementary Materials). An examination of the GO data obtained from enrichment in cellular components, biological processes and molecular function categories suggested that *Ah24* overexpression in Arabidopsis plants triggered mechanisms resembling those designed to re-establish and maintain ion and cellular homeostasis in response to stress (Conde et al., 2011). Particularly, relevant were the enrichment in categories involving stress sensing and signaling mechanisms, plant cell detoxification, transport and compartmentation. In this respect, the cellular component enrichment of the Golgi apparatus was relevant considering its vital role in the integration of plant growth and stress responses (Che et al., 2010). A possible influence of *Ah24* overexpression on developmental processers in Arabidopsis was suggested by the enrichment of various categories related to hormone responses, telomere maintenance and lengthening (Watson and Riha, 2010), histone modifications (Liu et al., 2010), carbon fixation and ribulose bisphosphate carboxylase activity, and energy-producing processes. In this respect, the enrichment of the 6-phosphofructokinase complex, part of which is involved in the glycolytic process and curiously, anoxia-related stress (Mithran et al., 2013), and of mitochondrial respiratory chain complex IV assembly categories was relevant. Other enriched development-associated categories were pollen development, lateral root development, anatomical structure formation involved in morphogenesis, and pollen sperm cell differentiation.

To confirm the results obtained from the microarray analysis, 19 genes which showed a 3.5 ≤ Z-score ≥ 3.5 were selected for qRT-PCR analysis. The results obtained (Figures S10A,B in Supplementary Materials), although not as contrasting as those reported in the microarray, corroborated their up- or down-regulated expression in response to the OE of the *Ah24* gene in *A. thaliana*. Moreover, the down-regulation of the *MiR398* gene was inversely correlated with the 3.5-fold increase in the expression levels of cytochrome C oxidase subunit V, one of its known target genes (Jones-Rhoades and Bartel, 2004; Figure S10B in Supplementary Materials). Interestingly, the majority of the genes whose expression was affected in the *Ah24* OE Arabidopsis plants are closely involved in phytohormone-related processes directly involved in plant growth and development (Table S4 in Supplementary Materials).

## Discussion

This study describes the characterization of a novel *A. hypochondriacus* gene of unknown function. The isolation of the *Ah24* complete cDNA, revealed that Ah24 is a small, 17.6 kDa, acidic (pI = 5.71), cytoplasm-localized protein having putative glycosylation and phosphorylation sites that could be important for its biological function (Figure 1 and Figure S2 in Supplementary Materials). Experimental data obtained in this study complemented previous evidence that strongly suggested that JA-inducible Ah24 could have a role in the wound response and/or defense against chewing insect herbivores (Navarro-Meléndez, 2009; Massange-Sánchez, 2011; Figure S1 in Supplementary Materials; see below).

A search for homologous protein or nucleotide sequences in all available databases yielded only two hits, in *A. cruentus* and *A. retroflexus*. The limited information available suggested that *Ah24* was possibly an “orphan” or taxonomically restricted gene, which usually lack an assignable function and have no resemblance to any other protein or gene outside a restricted lineage (Dassanayake et al., 2009, 2011; Oh et al., 2012). The prediction was supported by a subsequent search in recently, available transcriptomic data gathered by the “1000 Plant Initiative.” It indicated that the Ah24 protein has homology with a reduced group of proteins, most of which are restricted to species within the Caryophyllales order (Figures 2, 3). Interestingly, this plant order is characterized by an abundance of extremophytes (Flowers and Colmer, 2008; Oh et al., 2012). Accordingly, Ah24 homologs are found in *Salicornia europaea*, an halophyte that is widely distributed in coastal and inland salt marshes around the world (Fan et al., 2013; Ma et al., 2013) and in various *Atriplex* species, many of which are used for the remediation of saline or heavy-metal contaminated soils (Kachout et al., 2010 and references therein). It is also present in exceptionally stress tolerant plants such as *Kochia scoparia* (Kalinina et al., 2012 and references therein), *Spergularia media* (syn. *S. maritima*) (Flowers and Colmer, 2008), and *Dianthus* sp. Not surprisingly, these and other related species have been considered as potential sources of abiotic stress-resistance genes for crop improvement (Pathak et al., 2014).

Preliminary experimental data associated *Ah24* expression with the JA-regulated wound response in grain amaranth. This study provided further support for this function by showing that the *Ah24* gene underwent a very rapid, strong and stable induction, both local and systemic, in response to mechanical damage, leaf-chewing insect herbivory and MeJA treatment (Table 1). The higher resistance to *M. sexta* folivory in *Ah24* overexpressing transgenic tobacco plants (Figure 9A) was in agreement with the wound- and JA-responsive nature of this gene in grain amaranth and suggested an important role in the regulation of JA-dependent defense responses against insect herbivores in these plants. Additional evidence in favor of this gene's role in defense was the induction in transgenic OE tobacco plants of biochemical traits positively associated with the wound/insect herbivory response in several *Nicotiana* species (Halitschke and Baldwin, 2003; Lou and Baldwin, 2003; Steppuhn et al., 2004; Zavala et al., 2004; Steppuhn and Baldwin, 2007) and in other plants as well, including grain amaranth (Sánchez-Hernández et al., 2004; Mithöfer and Boland, 2012; Fürstenberg-Hägg et al., 2013). These included the accumulation of JA, nicotine, and foliar trypsin inhibitor activity (Figures 9B–D) Additional metabolomic data (Figure 10 and Figure S9 in Supplementary Materials) supported the herbivory-induced accumulation of both JA and nicotine, and further suggested that the accumulation of several other defense-related secondary metabolites, including alkaloids, phenolic compounds and phenolic amides, sesquiterpenes and diterpenoid glycosides (Nugroho and Verpoorte, 2002; Jassbi et al., 2008 and references therein; Großkinsky et al., 2012) was associated with the increased resistance to insect herbivory in transgenic tobacco.

Parallel experimental evidence gathered in amaranth and in transgenic OE Arabidopsis indicated that this gene might be also actively involved in the regulation of vegetative growth, particularly in developing tissues. The higher *Ah24* expression levels detected in apical meristems, young leaves and panicles compared to those in older tissues, such as mature leaves and stems of grain amaranth plants, supported this proposal (Figure 4A). A further role in leaf tissue regeneration after wounding was also implied by the strong *Ah24* expression detected in apical and axilar meristems and in the first leaves emerging in the early stages of recovery after severe defoliation in grain amaranth plants (Figures 4B,C). Thus, Ah24 may actively participate in the strong re-growth process observed in defoliated grain amaranth (Castrillón-Arbeláez et al., 2012; Vargas-Ortiz et al., 2013).

The above results suggested the participation of this gene both in wound-healing and leaf regeneration and in defense against leaf-chewing insect herbivory. However, an examination of the *Ah24* gene promoter region indicated a predominance of regulatory elements involved in development, thus providing a hint that this gene's role might be more oriented toward participation in developmental processes associated with leaf regrowth than in defense (Tables S1, S2 in Supplementary Materials). Particularly abundant were the AHBP, CCAF, L1, GT, and MADS boxes. These cis-acting elements are related to a wide range of functions, including maintenance of floral meristem identity and flower formation. They are also known to control the development of cotyledonary and vegetative tissue, fruit and/or roots, and to regulate epidermal cell differentiation and circadian factors that determine long-day photo-periodicity (Ng and Yanofsky, 2001; Harmer and Kay, 2005; Avilés-Arnaut and Délano-Frier, 2012; Takada and Iida, 2014). Their role in the response to abscisic acid stimulus has also been reported (Zhou, 1999; Venu et al., 2013).

Moreover, the phenotypes observed in transgenic *Ah24* OE Arabidopsis plants, which were gene-dosage dependent, further supported a role for the *Ah24* gene in development, as demonstrated by a temporary negative effect on root and inflorescence stem lengths (Figures 6, 7). These effects contrasted with increased vegetative growth, manifested later-on in development, as increased shoot biomass and leaf number (Figure 8). The microarray data (Table S3 in Supplementary Materials), together with a selection of the most highly induced and repressed genes (Table S4 in Supplementary Materials) showed that several genes directly involved in phytohormone-associated processes were induced in the very highly *Ah24* expressing L15 line. This pattern suggests that developmental changes observed in *Ah24* OE Arabidopsis could partly result from phytohormone-related developmental programs. However, the above described GO enrichment data (Table S3 in Supplementary Materials), imply that Ah24 influence on Arabidospis development might involve a more complex process, more akin to the mechanisms activated to re-establish and maintain ion and cellular homeostasis in response to stress (Conde et al., 2011). Apart from these conjectures, the mechanisms by which *Ah24* overexpression alters development in Arabidospis remain unknown. However, the predicted glycosylation and phosphorylation sites in its sequence could regulate its biological activity by shifting its predominant cytoplasmic localization to other organelles, including the nucleus (Figure 5), or could enhance its ability to form active complexes with developmentally relevant proteins (Blom et al., 2004).

On the other hand, as shown in Figure 5, Ah24 appears to accumulate predominantly around the nucleus, perhaps indicating that its biological activity could also be associated with this localization in the cell. This proposal is in agreement with a number of studies that have reported the significance that the nuclear surface/envelope has on cell division and cell elongation via its role as a microtubule organizing center from which the plasma membrane-cell wall continuum is generated. Also of relevance to this study is the proposed role played by this network in the regulation of plant responses to environmental stress conditions (Stoppin et al., 1994; Zhou et al., 2007; Ambrose and Wasteneys, 2014). Although many aspects of this network remain to be determined, it appears that many putative plasma membrane-cell wall anchoring proteins, similar to those identified in cell adhesion in other eukayotes, may serve as a transmembrane linkers between the extracellular matrix and the cytoskeleton to regulate plant growth, immune system responses and/or sensing of biotroph pathogens, cell wall mechanical tension or osmotic stress (Liu et al., 2015). Intriguingly, the GO enrichment data (Table S3 in Supplementary Materials) derived from the microarray assay revealed several biological process and molecular function categories that could contribute to this particular intracellular communication, including those representing microtubule polymerization or depolymerization, nucleologenesis, regulation of cell communication, and actomyosin structure organization. Moreover, an enrichment of GO cellular component categories possibly related to this network, such as those related to organelle envelope, or intrinsic to the membrane, microtubule and cell wall, was also found.

Interestingly, the development-associated effects observed in transgenic Arabidopsis plants were not mirrored in tobacco *Ah24* OE plants (Figure S8 in Supplementary Materials). On the other hand, the resistance to insect herbivory in transgenic tobacco plants was not reproduced in transgenic Arabidopsis plants, which showed no increased resistance to herbivory by *Trichoplusia ni* larvae (Figure S11 in Supplementary Materials). However, Arabidopsis *Ah24* OE plants appear to be strongly tolerant to water-stress (Massange-Sánchez, unpublished data), a finding which coincides with the frequent abiotic-stress resistance associated with “orphan-genes” such as this one (Oh et al., 2012). Perhaps the difference in *Ah24* gene dosage was a factor that contributed to the differences observed between transgenic Arabidopsis and tobacco plants (compare Figure S5 and Figure S7 in Supplementary Materials). For instance, the very highly expressing Arabidopsis L15 line had expression levels that were ca. 140-fold higher than those detected in the most actively *Ah24*-expressing tobacco L14 line. Conversely, it is not altogether unreasonable to suggest that the phylogenetic distance between these genera (Soltis et al., 1999) might have contributed to the different effects caused by the overexpression of the *Ah24* gene. The generation of similar phenotypes in transgenic Arabidospsis and tobacco overexpressing the same foreign gene is more frequent than not and is indicative of conserved gene functions in these genera (Rodrigues et al., 2006). However, the opposite has also been reported, even in closely related species such as tobacco and tomato. For example, the effect of overexpressing a key sterol biosynthetic gene from *Brassica juncea* had a much more positive effect in tobacco than in Arabidopsis (Liao et al., 2014). Moreover, different sensitivity to cytokinins was believed to cause the differential effects observed in transgenic tobacco and tomato transformed with the *CKI-1* gene, which is involved in this phytohormone's function (Mythili et al., 2011).

In conclusion, it appears that *Ah24* gene is involved in regulating growth in rapidly developing tissues of grain amaranth plants, such as meristems, young leaves, and panicles. This property, coupled to its strong induction by mechanical wounding, chewing insect herbivory and JA, is believed to be an important factor contributing to the plant's rapid recovery after severe defoliation, although a defensive function in grain amaranth cannot be ruled out. Despite the potential lack of biological relevance generated by experiments in which genes are expressed at non-physiological levels (Prelich, 2012), a role of the *Ah24* gene in both defense and development was, nevertheless, supported by data generated from the functional characterization of this gene in OE Arabidopsis and tobacco plants. However, the precise mechanism(s) by which Ah24 is able to modulate defense and development responses in these plants remain(s) unknown.

## Materials and methods

### Biological material

Seeds of *Amaranthus hypochondriacus* cv. Revancha were kindly provided by Dr. E. Espitia (INIFAP, México). Plants were maintained as described previously (Délano-Frier et al., 2011). *A. hypochondriacus* plants were used for experimentation 5–6 weeks after germination. At this point, they were 17–22 cm high and had 9–15 expanded leaves. Leaf, stem and root tissue samples were collected in all stress assays performed. Larvae of the Hawaiian beet webworm, *Spoladea recurvalis*, were employed for these herbivory experiments. Adult specimens were collected in the wild and reared in our laboratory: larvae fed on leaves of young amaranth plants and adults from cotton balls infused with diluted honey. *Manduca sexta* larvae were obtained from the insectary at Cinvestav, Irapuato. Tobacco (*Nicotiana tabacum* cv. Xanthi nc) and *Arabidopsis thaliana* ecotype Columbia were used for the generation of transgenic plants. *A. thaliana* seeds were germinated and grown following standard methodologies (http://web.calstatela.edu/faculty/vllnwth/grow.htm). Briefly, after a 72 h scarification-vernalization period at 4°C, sterilized seeds were germinated and grown on petri dishes containing Murashige and Skoog (MS; Murashige and Skoog, 1962) and then transferred to soil. Arabidospis plants were grown in controlled conditions of light (photosynthetic photon flux density (PPFD) of ≈200 μmol m^−2^ s^−1^), photoperiod (16 h light/8 h dark) and temperature (22 ± 1°C). Tobacco plants were grown as described previously (Valenzuela-Soto et al., 2011).

### Full-length cDNA and gene amplification

The full-length *Ah24* cDNA was obtained using total *A. hypochondriacus* leaf RNA (1 μg) as starting material. RNA was reverse-transcribed to generate the first-strand cDNA as described elsewhere (Castrillón-Arbeláez et al., 2012). Aliquots of this reaction (2 μl) were directly used as template for the PCR reactions in the presence of 100 pmol each of the specific primers Ah24-F and Ah24-R (Fomsgaard et al., 2010) (Table S5 in Supplementary Materials). Cloning and sequencing of the resulting amplicons were performed to confirm their identity. Subsequent amplification of the 5′ and 3′ cDNA ends was performed by RACE (Rapid Amplification of cDNA Ends) with the SMARTer RACE cDNA Amplification Kit (Clontech, Laboratories, Mountain View, CA), according to the manufacturer's instructions. The protein encoded by the open reading frame (ORF) of the *Ah24* cDNA was deduced with the aid of the FastPCR 6.0 program (http://en.bio-soft.net/pcr/FastPCR.html). Bioinformatic analyses were performed to determine the possible biological function of the Ah24 protein and to search for conserved domains. The following tools were used for this purpose: BlastN, BlastX and specialized Blast NCBI databases (http://blast.ncbi.nlm.nih.gov/), EMBL databases for InterProScan sequence searches and T-Coffee multiple alignment of sequences (http://www.ebi.ac.uk/embl/), and CBS databases to determine protein structure (CPHmodels 3.0 Server and CLC Main Workbench 6 Software), protein targeting (SignalP 3.0 Server), and protein localization (TargetP 1.1 Server) (http://www.cbs.dtu.dk/services/). ExPASy tools (http://www.expasy.ch/tools/) was used to predict secondary structures. The relationship of Ah24 with homologous proteins predicted from transcriptomes of exotic plant species, made available by the 1000 Plants initiative (https://sites.google.com/a/ualberta.ca/onekp/), was inferred by constructing a phylogenetic tree using MEGA Ver. 5.1 (Tamura et al., 2011).

Genomic DNA was extracted from leaves of *A. hypochondriacus* as previously instructed (Murray and Thompson, 1980) and digested with four restriction enzymes (DraI, EcoRV, PvuI, and StuI). The resulting fragments were blunt-end ligated to the Genome-Walker Adaptor provided by the Genome-Walker Universal kit (Clontech) to generate the corresponding libraries. These libraries were used as templates for PCR and nested PCR using primers based on the complete *Ah24* cDNA sequence (see Table S5 in Supplementary Materials). Amplifications in both 3′ and 5′ directions yielded the complete sequence of this gene, including a sizeable portion of its promoter region. The overlap between the cDNA and genomic sequences confirmed the identity of the newly generated fragments. All PCR amplicons obtained were cloned using the pCR4-TOPO cloning kit (Thermo Fisher Scientific, Waltham, MA USA) and sequenced. The intronic and exonic regions of the *Ah24* gene were defined using the ExonMapper Release 3.51 (Genomatix). A MatInspector Release professional 8.0.5 program (Genomatix), was used to detect regulatory elements in the 1304 bp promoter region of the *Ah24* gene.

### Stress treatments in *A. hypochondriacus*

#### Salt and water stress assays

Groups of six *A. hypochondriacus* plants were used to perform all stress experiments. These were performed in a growth chamber under controlled conditions of light (PPFD ≈300 μmol m^−2^ s^−1^) photoperiod and temperature (16 h light/8 h dark, 28°C). Plants were subjected to acute salt stress by watering the 1.3-L pots for three consecutive days with 40 mL of a 400 mM NaCl solution. Salt-treated plants (electrical conductivity of the run-off water ≈20 dS/m), showed little signs of stress compared to controls (0.28 dS/m in run-off water). The conductivity in the soil of salt-treated plants ranged between 2 and 3 dS/m, which are values that represent low to mild salinity conditions (http://www.fao.org/docrep/005/y4263e/y4263e0e.htm). Water stress, without acclimation (designed to measure basal tolerance), was imposed in the above conditions, by withholding irrigation for 5 days. Water-stressed plants showed visible signs of wilting and suffered partial leaf abscission.

#### Mechanical wounding, insect herbivory, methyl jasmonate (MeJA), and defoliation assays

Mechanical wounding was caused by the combined action of a nail cutter and a paper puncher. Using these implements, two large incisions on the edge of the leaves and two perforations in the central area of the leaf were made. Removed tissue amounted to 30–40% of the total leaf area. Only one leaf, placed mid-way to the apex of the plant, was damaged (usually leaf number 5, starting from bottom to top). Here, the local response to mechanical wounding was measured. The “systemic” wound response was determined in a younger leaf positioned at an angular distance of ca. 0° with respect to the damaged leaf (usually leaf number 9, starting from bottom to top). This sampling strategy was based on findings in other plant species (e.g., *N. attenuata*; Schittko and Baldwin, 2003; Orians, 2005), in which this phyllotactic pattern involves leaves that share primary vasculature. Sampling was performed at 1, 3, 6, 9, 12, 24, and 36 h after mechanical damage.

For the insect herbivory assays, three 3rd to 4th instar *S. recurvalis* caterpillars were confined to the older leaves of the bottom end of each plant by enclosing them within a plastic mesh sleeve by means of a clothespin. Leaves of undamaged control plants were also enclosed within plastic mesh sleeves. Larvae were allowed to feed for 3, 6, 9, 12, 24, and 36 h and were then removed. Insect herbivory damage caused approximately 30% of leaf surface loss. Both damaged (i.e., number 1–4, starting from the bottom) and undamaged (i.e., number 6–9, starting from the mid-section to the apex of the plant) leaves were collected from each plant and pooled into a single sample to determine the local and systemic herbivory-induced expression, respectively, of the *Ah24* gene.

MeJA treatments consisted of applying two drops of a 9.2 mM MeJA in 0.125% Triton X-100 (equivalent to 32 μg of MeJA per plant), to leaf number 5, as described above. Leaves number 5 (local response) and 9 (systemic response) (see above) were sampled 1, 3, 6, 9, 12, 24, and 36 h after MeJA application. Control plants were treated with the detergent solution only and were sampled at the same time points. Stem and root samples were also collected in each of the experiments described. All plant tissue sampled were flash frozen in liquid N_2_ and stored at −80°C until required for further analysis.

Severe defoliation of 45 days-old plants having approximately 11 expanded leaves was done mechanically as previously described (Vargas-Ortiz et al., 2015). Briefly, the complete loss of the plant's leaf tissue (except the remaining foliar tissue around the apical meristem), was completed within a 3-day period by eliminating 25% leaf tissue-loss per day by means of a punch-hole borer. On the third day, after 75% defoliation, plants were completely defoliated by cutting the remaining leaves at the base of the petiole. Apical meristems and the youngest leaf of the group of immature leaves remaining in the apex of the defoliated plants were collected immediately once defoliation was completed. Later, new leaves emerging from the axillary meristems were collected from the plants at 3, 6, and 9 days after defoliation. Leaves from developmentally matching intact plants were collected simultaneously to serve as controls.

### Quantitative PCR assays

All quantitative PCR (qPCR) assays were performed as described previously (Castrillón-Arbeláez et al., 2012; Casique-Arroyo et al., 2014) using SYBR Green detection chemistry and a CFX96 Real Time System (Bio-Rad, Hercules, CA, USA). qPCR reactions were performed using the *Ah24* primers listed in Table S5 of Supplementary Materials, which were designed on the basis of its complete cDNA sequence. The *Ah24* expression levels in different *A. hypochondriacus* plant tissues (i.e., young, and intermediate leaves, stems from young and mature plants, roots, apical and axillar meristems and panicles) were reported in relation to the expression levels in mature leaves, which were set at a value of 1 (Ying et al., 2012). In transgenic Arabidopsis and tobacco plants, foliar *Ah24* transgene expression levels were calculated relative to trace background levels in WT plants. Relative gene expression was calculated using the comparative cycle threshold method (Livak and Schmittgen, 2001). The genes employed as normalizing controls were actin and tubulin (for amaranth), actin and *EF-1*α (for Arabidopsis) and the *L25 Ribosomal protein* gene and *EF-1*α (for tobacco). In all cases, plant tissues for qPCR assays were obtained from combined pools of six plants. Each pool was subsequently subjected to three independent sampling procedures prior to analysis. qPCR data are reported as the mean of three repetitions ± SE of one representative experiment. qRT-PCR expression analyses were validated in two independent experiments.

Primers employed for the validation of the microarray assay performed in *Ah24* overexpressing *A. thaliana* plants (see below) are also shown in Table S5 of Supplementary Materials. Primer design for *Ah24* and selected Arabidopsis genes was performed using DNA calculator software (Sigma-Aldrich St. Louis, MO, USA) and included part of the unique 3′ non-coding region to ensure specificity.

### Plant transformation

#### Overexpression construct and floral dip transformation of arabidopsis

The complete ORF of *Ah24* was PCR amplified using specific primers (Table S5 in Supplementary Materials). The amplified 579 bp fragment was cloned in the pCR 8/GW/TOPO TA entry vector (Thermo Fisher Scientific) and subsequently in the pB7WG2D exit vector (Cambia, Canberra, Australia), under the control of 35S CaMV promoter. In both cases, spectinomycin resistance was used for selection in *E. coli*. The binary construct was electroporated into *Agrobacterium tumefaciens* strain GV2260. *A. thaliana* plants were transformed by the floral dip method (Clough and Bent, 1998). Dipped plants were grown to maturity under controlled conditions, as described above, and the seeds were harvested at maturity. Two-week old T1 OE transgenic *A. thaliana* plants were selected by growth in MS medium containing N-acetyl-L-phosphinothricin (PPT) (10 μg/mL) and screening by PCR using gene-specific primers (Table S5 in Supplementary Materials). T2 seeds were collected from individual transformants (T1) and plated again on the selection medium to determine PPT-resistant vs. PPT-sensitive plants segregation ratios. Homozygous transgenic plants produced no PPT-sensitive seedlings from seeds of T_2_ plants. All further analysis of *Ah24* overexpressing (OE) lines was performed using T_2_ plants homozygous for the transgene.

Seeds of WT controls and homozygous OE transgenic (T2) Arabidopsis lines differing in *Ah24* transgene dosage (i.e., L5, L11, and L15), were germinated in petri dishes containing MS salt medium as above. Eight days later, the germinated seedlings were transferred to 40-well trays or single 125 mL plastic pots containing a soil mixture composed of three parts Sunshine Mix 3TM (SunGro Horticulture, Bellevue, WA), one part loam, two parts mulch, one part vermiculite (SunGro Hort), and one part perlite (Termolita S.A., Nuevo León, México). The Arabidopsis plants were grown in controlled conditions, as above. Plants were fertilized once with 2.0 g/L of a 20-10-20 (N-P-K) all-purpose plant fertilizer (Peters Professional; Scotts-Sierra Horticultural Products, Marysville, OH, USA) just after seedlings were transferred to the plastic pots. Plants intended for seed production were fertilized once more at the onset of flowering. In these conditions, the life cycle of the Arabidopsis plants from planting to harvest of seeds was ca. 8 weeks. Several independent sets of OE transgenic and wild-type plants were grown for the different analyses performed. Four weeks-old plants were used for experimentation.

#### Construction of plant transformation vector and tobacco transformation

To perform plant transformation, *Ah24* cDNA was PCR-amplified with a primer pair listed in Table S5 of Supplementary Materials. These amplified an 810 bp fragment, which was cloned into the pMDC32 transformation vector and placed under the control of the 2 × 35 S CaMV promoter, in tandem. The resulting vector was mobilized into *A. tumefaciens* (LBA4404; Voelker et al., 1987) and used to transform tobacco plants according to a standard protocol (Avilés-Arnaut and Délano-Frier, 2012).

Briefly, sterile tobacco seeds were germinated on 0.5 × MS medium, containing 1% sucrose and 1% phytagar (Thermo-Fisher Scientific), respectively. Leaf segments cut from the germinated plants were used for transformation with *A. tumefaciens.* Putative OE transgenic plants were regenerated directly from leaf edges in the presence of hygromycin B (HygB; 25 mg/L). These were grown in a conditioned growth chamber maintained at 28°C with a 16 h/8 h light photoperiod and a PPFD of ≈300 μmol m^−2^ s^−1^. Seeds from 18 parental T0 OE transgenic plants were germinated to generate T1 generation plants. Six plants from each resulting T1 line were selected at random and 200 seeds from each were germinated on MS- HygB media to select plants having a single copy of the gene. Transgenic OE plants were screened by PCR using the HygB -selective primers (Table S5 in Supplementary Materials), which generated a 163 bp amplicon. Three T2 lines with different *Ah24* transgene dosage (i.e., L15, L2, and L14) showing 100% survival on the HygB-supplemented (20 μg/mL) MS medium and having no abnormal phenotypes were selected for further studies. Transgenic OE tobacco plants were subsequently grown under controlled conditions in the above growth chamber or in a greenhouse, under natural conditions of light temperature present during the spring-summer of 2014.

### Plant growth, leaf number, seed capsule and flower counts in transgenic tobacco plants

WT plant seeds or of the three transgenic OE lines selected were germinated in petri dishes containing MS media ± HygB, as above. Approximately 1 week later, seedlings were transferred to germinating trays containing a germination soil mixture, as described previously (Casique-Arroyo et al., 2014). Approximately 3-weeks later, young plantlets were transferred to 4-L plastic pots containing a general soil mixture (Casique-Arroyo et al., 2014) and were grown in a greenhouse, under natural conditions of light and temperature present during the spring-summer of 2014. After 28 days, plant height and leaf number were determined weekly in ≈8 weeks-old plants per genotype (*n* = 12). Flowers were counted weekly once flowering started, which occurred ≈25 days later. For seed production, capsules were collected from the plants as they matured. The number of flower and seed capsules, as well as total seed biomass were considered as fitness components.

### Measurement of vegetative growth and seed yield in transgenic arabidospis plants

The effect on vegetative growth and seed yield was determined in three transgenic OE Arabidopsis plants lines differentially overexpressing the *Ah24* gene. Root growth was determined at different developmental stages: in 2, 3, 6, 8, and 9 days-old seedlings grown vertically on MS-containing petri dishes and in 6–7 weeks-old plants grown in soil. Leaf number as well as fresh and dry weights of rosettes, roots and inflorescences was also determined in these plants. To measure yield, dry seeds were harvested manually from fully matured plants, approximately after 8 weeks of growth. All experiments were performed in the Arabidopsis growth chamber, as described above.

### Construction of the GFP::Ah24 fusion construct and transformation of A. thaliana

The coding sequence of the *Ah24* gene, was fused with the 5′ region of green fluorescent protein (GFP) driven by the 35S CaMV promoter. The PCR product was amplified with specific primers listed in Table S5 of Supplementary Materials, and was subsequently cloned into the pDONR 221 plasmid and transferred to the pFAST-R06 plasmid using the Gateway technology (Invitrogen, Carlsbad, CA, USA). The binary vector was electroporated inro *E*. *coli* (DH5α strain) and *A. tumefaciens* (GV2260 strain). Arabidopsis plants (Col-0) were transformed with *Agrobacterium* using a modified floral dip method, as above. For GFP analysis, roots were observed under an inverted LSM510 confocal laser scanning microscope (Zeiss, Oberkochen, Germany). For visualization, seedlings were stained and mounted in 10 μg/ml propidium iodide (PI) solution (Sigma). GFP was excited with the 488 nm laser line of an argon laser, whereas PI was excited with the 514 nm laser line. The resulting images were acquired using the multi-channel mode. For GFP analysis of whole seedlings, a Lumar V.12 stereoscopic microscope with a GFP filter (Zeiss) was used.

### Insect herbivory bioassays in transgenic tobacco

The herbivory assays in transgenic OE tobacco plants were performed in 8-weeks-old plants having 10–14 expanded leaves. Groups of six plants comprising WT and 3 T2 transgenic OE lines, respectively, were challenged with two 2nd or 3rd instar *M. sexta* larvae per plant, specifically in leaves 7 and 8. The experiments were performed in a conditioned growth chamber maintained as described above. *M. sexta* larvae fed on the plants for 3 days. Resistance to insect herbivory was measured as a reduction in larval body weight gain, a commonly used parameter to measure antifeedant effects in plants, by deducting the initial body weight from the final body weight of each larva. Damaged leaves (“local” response; leaves 7 and 8) and distal, undamaged leaves (“systemic” response; leaves 11–14) from two plants were pooled into three groups for analysis. Data collected from two independent experiments yielded similar results. Thus, only data from a representative experiment were reported.

### Measurement of defense-related parameters in transgenic tobacco subjected to insect herbivory

Trypsin inhibitor levels of activity were determined according to a reported method (Erlanger et al., 1961). The technique was adapted to fit a microplate format. Activity was expressed as units of inhibitory activity per mg of protein. Protein was determined according to the Bradford method (Bradford, 1976) employing a commercial kit (Bio-Rad). Nicotine levels were determined by GC/MS as described previously (Hossain and Salehuddin, 2013). JA levels were also determined by GC/MS using previously reported methodologies for extraction (Pluskota et al., 2007) and derivatization prior to GC/MS analysis (Mueller and Brodschelm, 1994). A full spectrum of ionizable metabolites were also measured using direct injection electron spray ionization mass spectrometry (DIESI-MS) according to previous reports (García-Flores et al., 2012; Montero-Vargas et al., 2013), following optimized protocols in data acquisition and bioinformatic data processing (Garcia-Flores et al., unpublished data). Briefly, finely sieved (300 μm particle size mesh) lyophilized leaf powder was extracted with acidified HPLC grade methanol at 25°C for 2 h with constant agitation. Filtered extracts (0.45 μm) were injected to a SQ2 quadrupole mass spectrometer (Waters Corp., Milford, MA, USA). Ions were measured both in negative and positive ion mode for a period of 60 s each and intensity data was averaged and then analyzed according to Garcia-Flores et al. (unpublished data). The raw MS spectra files were converted to ^*^.mzXML using masswolf V 1.4. Analysis of mass spectra was performed with the OpenMS/TOPP suite, version 1.8.0 and further processed with the free statistical R software (http://www.r-project.org).

### Microarray analysis

Microarray analysis were performed at the Microarray Unit of the Cellular Physiology Institute of the National Autonomous University of Mexico (UNAM). The analysis was performed with rosette leaves of 4-to-5-week-old WT and transgenic *Ah24* OE Arabidopsis plants. For array printing, an *A. thaliana* 70-mer oligo library from OPERON Oligo Sets (http://omad.operon.com/) was re-suspended to a final concentration of 40 μM in Micro Spotting solution (TeleChem International Inc., Sunnyvale, CA, USA). SuperAmine coated slides 25 × 75 mm (TeleChem International Inc.) were printed in single copy, and fixed at 80°C for 4 h. For pre-hybridization the slides were rehydrated with water vapor at 60°C, and fixed with two cycles of UV light (1200 J). After boiling for 2 min at 92°C, slides were washed with 95% ethanol for 1 min and pre-hybridzed in 5 × SSC, 0.1% SDS and 1% BSA for 1 h at 42°C. The slides were washed and dried for further hybridization. The printing geometry employed was amenable to the GEO platform, consisting of grid “X” and “Y” coordinates in slide of 7 and 5.5 mm, dot spacing “Y” 170 microns, dot spacing “X” 170 microns, number of dots per grid “X” and “Y” 25, distance between grids “X” and “Y” 150 microns, and number of grids 48. For probe preparation and hybridization to arrays, 10 μg of total RNA were used for a cDNA synthesis procedure that incorporated either dUTP-Alexa555 or dUTP-Alexa647. This was performed employing the First-Strand cDNA labeling kit (Invitrogen). Incorporation of the fluorophore was analyzed at 555 and 650 nm for Alexa555 and Alexa647, respectively. Equal quantities of labeled cDNA were hybridized using UniHyb hybridization solution (TeleChem International Inc.). The arrays were incubated for 14 h at 42°C, and then washed tree times with 1 × SCC, 0.05% SDS at room temperature. Data acquisition and quantification of array images was performed in ScanArray 4000 with its accompanying software ScanArray 4000 from Packard BioChips (Packard Biochip Technologies, LLC, Billerica, MA, USA). All images were captured using 65% PMT gain, 70–75% laser power and 10 μm resolution at 50% scan rate. For each spot the Cy3 and Cy5 density mean value and the Cy3 and Cy5 background mean value were calculated with an ArrayPro Analyzer software (Media Cybernetics, Rockville, MD, USA). Microarray data analysis was performed with the free software genArise, developed in the Computing Unit of the Cellular Physiology Institute at UNAM (http://www.ifc.unam.mx/genarise/). GenArise performs a number of transformations: background correction, lowess normalization, intensity filter, replicates analysis, and selection of differentially expressed genes. GenArise is designed to identify which of the genes show strong evidence of being differentially expressed. The software identifies differential expressed genes by calculating an intensity-dependent z-score. This is done using a sliding window algorithm to calculate the mean and standard deviation within a window surrounding each data point, in order to define a z-score where z measures the number of standard deviations a data point is from the mean.

$$\begin{array}{l} z_{i}=\left(R_{i}--mean\left(R\right)\right)∕sd\left(R\right) \end{array}$$

Where z_i_ is the z-score for each element, R_i_ is the log-ratio for each element, and sd(*R*) is the standard deviation of the log-ratio. With this criterion, only the elements with an absolute Z-score greater than 2 were considered differentially expressed (Z-score > 2; Cheadle et al., 2003). The elements with a z-score > 2 standard deviations can be considered to be significantly differentially expressed genes.

The GO-term enrichment analysis was done through the PlantGSEA (Yi et al., 2013) platform, using exclusively the differentially expressed genes and the whole Arabidopsis genome as background. Enriched GO terms were selected based on a multiple test corrected (MTC) *P*-value (Bonferroni pval) < 0.05.

Microarray data was deposited as GEO accession GSE70272.

### Statistical analysis

All statistical analyses of the physiological and biochemical data were done using JMP8 at the α = 0.05 level (SAS Institute Inc., Cary, NC). Data were analyzed using an ANOVA. A Tukey test was performed with each ANOVA. In all figures, mean values and vertical bars representing standard errors (SE) are shown.

### Conflict of interest statement

The authors declare that the research was conducted in the absence of any commercial or financial relationships that could be construed as a potential conflict of interest.

## References

[B1] Aguilar-Hernández H. S. Santos L. León-Galván F. Barrera-Pacheco A. Espitia-Rangel E. De León-Rodríguez A.. (2011). Identification of calcium stress induced genes in amaranth leaves through suppression subtractive hybridization. J. Plant Physiol. 168, 2102–2109. 10.1016/j.jplph.2011.06.006 21794947

[B2] Ambrose C. Wasteneys G. O. (2014). Microtubule initiation from the nuclear surface controls cortical microtubule growth polarity and orientation in *Arabidopsis thaliana*. Plant Cell Physiol. 55, 1636–1645. 10.1093/pcp/pcu094 25008974 PMC4160572

[B3] Ariizumi T. Lawrence P. K. Steber C. M. (2011). The role of two F-Box proteins, SLEEPY1 and SNEEZY, in Arabidopsis gibberellin signaling. Plant Physiol. 155, 765–775. 10.1104/pp.110.166272 21163960 PMC3032465

[B4] Avilés-Arnaut H. Délano-Frier J. P. (2012). Characterization of the tomato prosystemin promoter: organ-specific expression, hormone specificity and methyl jasmonate responsiveness by deletion analysis in transgenic tobacco plants. J. Integr. Plant Biol. 54, 15–32. 10.1111/j.1744-7909.2011.01084.x 22044436

[B5] Bao F. Azhakanandam S. Franks R. G. (2010). SEUSS and SEUSS-LIKE transcriptional adaptors regulate floral and embryonic development in Arabidopsis. Plant Physiol. 152, 821–836. 10.1104/pp.109.146183 20007451 PMC2815852

[B6] Barah P. Winge P. Kusnierczyk A. Tran D. H. Bones A. M. (2013). Molecular signatures in *Arabidopsis thaliana* in response to insect attack and bacterial infection. PLoS ONE 8:e58987. 10.1371/journal.pone.0058987 23536844 PMC3607608

[B7] Blom N. Sicheritz-Pontén T. Gupta R. Gammeltoft S. Brunak S. (2004). Prediction of post-translational glycosylation and phosphorylation of proteins from the amino acid sequence. Proteomics 4, 1633–1649. 10.1002/pmic.200300771 15174133

[B8] Bradford M. (1976). A rapid and sensitive method for the determination of microgram quantities of protein utilizing the principle of protein dye-binding. Anal. Biochem. 72, 248–252. 10.1016/0003-2697(76)90527-3 942051

[B9] Brenner D. Baltensperger D. Kulakow P. Lehmann J. Myers R. Slabbert M. (2000). Genetic resources and breeding of *Amaranthus*. Plant Breed. Rev. 19, 227–285. 10.1002/9780470650172.ch7

[B10] Casarrubias-Castillo K. Martínez-Gallardo N. A. Délano-Frier J. P. (2014). Treatment of *Amaranthus cruentus* with chemical and biological inducers of resistance has contrasting effects on fitness and protection against compatible Gram positive and Gram negative bacterial pathogens. J. Plant Physiol. 171, 927–939. 10.1016/j.jplph.2014.02.004 24913050

[B11] Casique-Arroyo G. Martínez-Gallardo N. González de la Vara L. Délano-Frier J. P. (2014). Betacyanin biosynthetic genes and enzymes are differentially induced by (a)biotic stress in *Amaranthus hypochondriacus*. PLoS ONE 9:e99012. 10.1371/journal.pone.0099012 24896616 PMC4045864

[B12] Castrillón-Arbeláez P. A. Martínez-Gallardo N. Avilés-Arnaut H. Tiessen A. Délano-Frier J. P. (2012). Metabolic and enzymatic changes associated with carbon mobilization, utilization and replenishment triggered in grain amaranth (*Amaranthus cruentus*) in response to partial defoliation by mechanical injury or insect herbivory. BMC Plant Biol. 12:163. 10.1186/1471-2229-12-163 22966837 PMC3515461

[B13] Che P. Bussell J. D. Zhou W. Estavillo G. M. Pogson B. J. Smith S. M. (2010). Signaling from the endoplasmic reticulum activates brassinosteroid signaling and promotes acclimation to stress in Arabidopsis. Sci. Signal. 3, ra69. 10.1126/scisignal.2001140 20876872

[B14] Cheadle C. Vawter M. P. Freed W. J. Becker K. G. (2003). Analysis of microarray data using Z score transformation. J. Mol. Diagn. 5, 73–81. 10.1016/S1525-1578(10)60455-2 12707371 PMC1907322

[B15] Clough S. J. Bent A. F. (1998). Floral dip: a simplified method for *Agrobacterium*-mediated transformation of *Arabidopsis thaliana*. Plant J. 16, 735–743. 10.1046/j.1365-313x.1998.00343.x 10069079

[B16] Conde A. Chaves M. M. Gerós H. (2011). Membrane transport, sensing and signaling in plant adaptation to environmental stress. Plant Cell Physiol. 52, 1583–1602. 10.1093/pcp/pcr107 21828102

[B17] Curaba J. Singh M. B. Bhalla P. L. (2014). miRNAs in the crosstalk between phytohormone signaling pathways. J. Exp. Bot. 65, 1425–1438. 10.1093/jxb/eru002 24523503

[B18] Cutcliffe J. W. Hellmann E. Heyl A. Rashotte A. M. (2011). CRFs form protein–protein interactions with each other and with members of the cytokinin signalling pathway in Arabidopsis via the CRF domain. J. Exp. Bot. 62, 4995–5002. 10.1093/jxb/err199 21705390 PMC3193008

[B19] Dassanayake M. Haas J. S. Bohnert H. J. Cheeseman J. M. (2009). Shedding light on an extremophile lifestyle through transcriptomics. New Phytol. 183, 764–775. 10.1111/j.1469-8137.2009.02913.x 19549131

[B20] Dassanayake M. Oh D. H. Haas J. S. Hernandez A. Hong H. Ali S.. (2011). The genome of the extremophile crucifer *Thellungiella parvula*. Nat. Genet. 43, 913–918. 10.1038/ng.889 21822265 PMC3586812

[B21] Délano-Frier J. P. Avilés-Arnaut H. Casarrubias-Castillo K. Casique-Arroyo G. Castrillón-Arbeláez P. A. Herrera-Estrella L.. (2011). Transcriptomic analysis of grain amaranth (*Amaranthus hypochondriacus*) using 454 pyrosequencing: comparison with *A. tuberculatus*, expression profiling in stems and in response to biotic and abiotic stress. BMC Genomics 12:363. 10.1186/1471-2164-12-363 21752295 PMC3146458

[B22] Délano-Frier J. P. Martínez-Gallardo N. A. Martínez-de la Vega O. Salas-Araiza M. D. Barbosa-Jaramillo E. R. Torres A.. (2004). The effect of exogenous jasmonic acid on induced resistance and productivity in amaranth (*Amaranthus hypochondriacus*) is influenced by environmental conditions. J. Chem. Ecol. 30, 1001–1034. 10.1023/B:JOEC.0000028464.36353.bb 15274445

[B23] Erlanger B. Kokowsky N. Cohen W. (1961). The preparation and properties of two new chromogenic substrates of trypsin. Arch. Biochem. Biophys. 95, 271–278. 10.1016/0003-9861(61)90145-X 13890599

[B24] Fan P. Nie L. Jiang P. Feng J. Lv S. Chen X.. (2013). Transcriptome analysis of *Salicornia europaea* under saline conditions revealed the adaptive primary metabolic pathways as early events to facilitate salt adaptation. PLoS ONE 8:e80595. 10.1371/journal.pone.0080595 24265831 PMC3827210

[B25] Flowers T. J. Colmer T. D. (2008). Salinity tolerance in halophytes. New Phytol. 179, 945–963. 10.1111/j.1469-8137.2008.02531.x 18565144

[B26] Fomsgaard I. Añon M. Barba de la Rosa A. Christophersen C. Dusek K. Délano-Frier J. (2010). Adding Value to Holy Grain: Providing the Key Tools for the Exploitation of Amaranth - the Protein-Rich Grain of the Aztecs. Results from a Joint European - Latin American Research Project. Denmark: Department of Integrated Pest Management, Aarhus University, Faculty of Agricultural Sciences.

[B27] Fürstenberg-Hägg J. Zagrobelny M. Bak S. (2013). Plant defense against insect herbivores. Int. J. Mol. Sci. 14, 10242–10297. 10.3390/ijms140510242 23681010 PMC3676838

[B28] García-Flores M. Juárez-Colunga S. Montero-Vargas J. M. LÓpez-Arciniega J. A. I. Chagolla A. Tiessen A.. (2012). Evaluating the physiological state of maize (*Zea mays* L.) plants by direct-injection electrospray mass spectrometry (DIESI-MS). Mol. Biosyst. 8, 1658–1660. 10.1039/c2mb25056j 22513980

[B29] Giacomini D. Westra P. Ward S. M. (2014). Impact of genetic background in fitness cost studies: an example from glyphosate-resistant Palmer amaranth. Weed Sci. 62, 29–37. 10.1614/WS-D-13-00066.1

[B30] Grobelnik-Mlakar S. Bavec M. Jakop M. Bavec F. (2012). The effect of drought occurring at different growth stages on productivity of grain amaranth *Amaranthus cruentus*. G6. J. Life Sci. 6, 283–286.

[B31] Großkinsky D. K. van der Graaff E. Roitsch T. (2012). Phytoalexin transgenics in crop protection-Fairy tale with a happy end? Plant Sci. 195, 54–70. 10.1016/j.plantsci.2012.06.008 22920999

[B32] Halitschke R. Baldwin I. T. (2003). Antisense LOX expression increases herbivore performance by decreasing defense responses and inhibiting growth-related transcriptional reorganization in *Nicotiana attenuata*. Plant J. 36, 794–807. 10.1046/j.1365-313X.2003.01921.x 14675445

[B33] Harmer S. L. Kay S. A. (2005). Positive and negative factors confer phase-specific circadian regulation of transcription in Arabidopsis. Plant Cell 17, 1926–1940. 10.1105/tpc.105.033035 15923346 PMC1167542

[B34] Hossain A. M. Salehuddin S. M. (2013). Analytical determination of nicotine in tobacco leaves by gas chromatography-mass spectrometry. Arab. J. Chem. 6, 275–278. 10.1016/j.arabjc.2010.10.006

[B35] Huerta-Ocampo J. A. Barrera-Pacheco A. Mendoza-Hernández C. S. Espitia-Rangel E. Mock H. P. Barba de la Rosa A. P. (2014). Salt stress-induced alterations in the root proteome of *Amaranthus cruentus* L. J. Proteome Res. 13, 3607–3627. 10.1021/pr500153m 24942474

[B36] Huerta-Ocampo J. A. Briones-Cerecero E. P. Mendoza-Hernandez G. De Leon-Rodriguez A. Barba de la Rosa A. P. (2009). Proteomic analysis of amaranth (*Amaranthus hypochondriacus* L.) leaves under drought stress. Int. J. Plant Sci. 170, 990–998. 10.1086/605119

[B37] Huerta-Ocampo J. A. LeÓn-Galván M. F. Ortega-Cruz L. B. Barrera-Pacheco A. De León-Rodríguez A. Mendoza-Hernández G.. (2011). Water stress induces up-regulation of DOF1 and MIF1 transcription factors and down-regulation of proteins involved in secondary metabolism in amaranth roots (*Amaranthus hypochondriacus* L.). Plant Biol. 13, 472–482. 10.1111/j.1438-8677.2010.00391.x 21489098

[B38] Jassbi A. R. Gase K. Hettenhausen C. Schmidt A. Baldwin I. T. (2008). Silencing geranylgeranyl diphosphate synthase in *Nicotiana attenuata* dramatically impairs resistance to tobacco hornworm. Plant Physiol. 146, 974–986. 10.1104/pp.107.108811 17965175 PMC2259063

[B39] Johnson B. L. Henderson T. L. (2002). Water use patterns of grain amaranth in the northern Great Plains. Agron. J. 94, 1437–1443. 10.2134/agronj2002.1437

[B40] Jones-Rhoades M. W. Bartel D. P. (2004). Computational identification of plant microRNAs and their targets, including a stress-induced miRNA. Mol. Cell 14, 787–799. 10.1016/j.molcel.2004.05.027 15200956

[B41] Kachout S. S. Mansoura A. B. Leclerc J. C. Mechergui R. Rejeb M. N. Ouerghi Z. (2010). Effects of heavy metals on antioxidant activities of: *Atriplex hortensis* and *A. rosea*. Electron. J. Environ. Agric. Food Chem. 9, 444–457.

[B42] Kalinina E. B. Keith B. K. Kern A. J. Dyer W. E. (2012). Salt- and osmotic stress-induced choline monooxygenase expression in *Kochia scoparia* is ABA-independent. Biol. Plant. 56, 699–704. 10.1007/s10535-012-0132-0

[B43] Li J. Wang S. Liu X. Li X. L. Guo J. (1989). An observation of the root system growth of grain amaranth and its drought resistance. Agric. Res. Arid Areas 3, 34–41.

[B44] Liao P. Wang H. Wang M. Hsiao A. S. Bach T. J. Chye M. L. (2014). Transgenic tobacco overexpressing *Brassica juncea* HMG-CoA Synthase 1 shows increased plant growth, pod size and seed yield. PLoS ONE 9:e98264. 10.1371/journal.pone.0098264 24847714 PMC4029903

[B45] Liu C. Lu F. Cui X. Cao X. (2010). Histone methylation in higher plants. Ann. Rev. Plant Biol. 61, 395–420. 10.1146/annurev.arplant.043008.091939 20192747

[B46] Liu Z. Persson S. Sánchez-Rodríguez C. (2015). At the border: the plasma membrane–cell wall continuum. J. Exp. Bot. 66, 1553–1563. 10.1093/jxb/erv019 25697794

[B47] Livak K. J. Schmittgen T. D. (2001). Analysis of relative gene expression data using real-time quantitative PCR and the 2^−ΔΔCt^ method. Methods 25, 402–408. 10.1006/meth.2001.1262 11846609

[B48] Lou Y. Baldwin I. T. (2003). *Manduca sexta* recognition and resistance among allopolyploid *Nicotiana* host plants. Proc. Natl. Acad. Sci. U.S.A. 100, 14581–14586. 10.1073/pnas.2135348100 14530394 PMC304122

[B49] Ma J. Zhang M. Xiao X. You J. Wang J. Wang T.. (2013). Global transcriptome profiling of *Salicornia europaea* L. shoots under NaCl Treatment. PLoS ONE 8:e65877. 10.1371/journal.pone.0065877 23825526 PMC3692491

[B50] Massange-Sánchez J. A. (2011). Análisis de la Expresión y Caracterización Molecular del Gen Ah24 Inducido por Daño Mecánico, Herbivoría y Adición Exógena de MeJA en Plantas de Amaranthus Hypochondriacus. M.Sc. dissertation, Centro de investigación y de Estudios Avanzados del IPN, Unidad Irapuato.

[B51] Matsushita A. Furumoto T. Ishida S. Takahashi Y. (2007). AGF1, an AT-Hook protein, is necessary for the negative feedback of *AtGA3ox1* encoding GA 3-Oxidase1. Plant Physiol. 143, 1152–1162. 10.1104/pp.106.093542 17277098 PMC1820926

[B52] Memelink J. (2009). Regulation of gene expression by jasmonate hormones. Phytochemistry 70, 1560–1570. 10.1016/j.phytochem.2009.09.004 19796781

[B53] Miller T. E. Wing J. S. Huete A. R. (1984). The agricultural potential of selected C4 plants in arid environments. J. Arid Environ. 7, 275–286.

[B54] Mithöfer A. Boland W. (2012). Plant defense against herbivores: chemical aspects. Annu. Rev. Plant Biol. 63, 431–450. 10.1146/annurev-arplant-042110-103854 22404468

[B55] Mithran M. Paparelli E. Novi G. Perata P. Loreti E. (2013). Analysis of the role of the pyruvate decarboxylase gene family in *Arabidopsis thaliana* under low-oxygen conditions. Plant Biol. (Stuttg) 16, 28–34. 10.1111/plb.12005 23574450

[B56] Montero-Vargas J. M. González-González L. H. Gálvez-Ponce E. Ramírez-Chávez E. Molina-Torres J. Chagolla A.. (2013). Metabolic phenotyping for the classification of coffee trees and the exploration of selection markers. Mol. Biosyst. 9, 693–699. 10.1039/c3mb25509c 23385826

[B57] Mueller M. J. Brodschelm W. (1994). Quantification of jasmonic acid by capillary gas chromatography-negative chemical-ionization mass-spectrometry. Anal. Biochem. 218, 425–435. 10.1006/abio.1994.1202 8074303

[B58] Murashige T. Skoog F. (1962). A revised medium for rapid growth and bio assays with tobacco tissue cultures. Physiol. Plant. 15, 473–497. 10.1111/j.1399-3054.1962.tb08052.x

[B59] Murray M. G. Thompson W. F. (1980). Rapid isolation of high molecular-weight plant DNA. Nucleic Acids Res. 8, 4321–4325. 10.1093/nar/8.19.4321 7433111 PMC324241

[B60] Mythili J. B. Saiprasad G. V. Naveena C. Rajeev P. R. Upreti K. K. (2011). Differential response of tomato and tobacco to Agrobacterium mediated transformation with cytokinin independent-1 (*CKI-1*) gene as influenced by cytokinin levels. Indian J. Exp. Bot. 49, 901–918. 22403864

[B61] Navarro-Meléndez A. L. (2009). Análisis Proteómico del Amaranto, Sometido a Herbivoría (Spodoptera exigua) y/o Evocadores Relacionados con la Resistencia a Insectos, MeJA. M.Sc. dissertation, Centro de investigación y de Estudios Avanzados del IPN, Unidad Irapuato.

[B62] Ng M. Yanofsky M. F. (2001). Function and evolution of the plant MADS-box gene family. Nat. Rev. Genet. 2, 186–195. 10.1038/35056041 11256070

[B63] Niveyro S. Salvo A. (2014). Taxonomic and functional structure of phytophagous insect communities associated with grain amaranth. Neotrop. Entomol. 43, 532–540. 10.1007/s13744-014-0248-3 27194061

[B64] Nugroho L. H. Verpoorte R. (2002). Secondary metabolism in tobacco. Plant Cell Tiss. Org. 68, 105–125. 10.1023/A:1013853909494

[B65] Oh D. H. Dassanayake M. Bohnert H. J. Cheeseman J. M. (2012). Life at the extreme: lessons from the genome. Genome Biol. 13, 241. 10.1186/gb4003 22390828 PMC3439964

[B66] Omami E. N. Hammes P. S. Robbertse P. J. (2006). Differences in salinity tolerance for growth and water−use efficiency in some amaranth (*Amaranthus* spp.) genotypes. NZ. J. Crop Hort. Sci. 34, 11–22. 10.1080/01140671.2006.9514382

[B67] Orians C. (2005). Herbivores, vascular pathways, and systemic induction: facts and artifacts. J. Chem. Ecol. 31, 2231–2242. 10.1007/s10886-005-7099-7 16195841

[B68] Pathak M. R. Teixeira da Silva J. A. Wani S. H. (2014). Polyamines in response to abiotic stress tolerance through transgenic approaches. GM Crops Food 5, 87–96. 10.4161/gmcr.28774 24710064 PMC5033173

[B69] Pluskota W. E. Qu N. Maitrejean M. Boland W. Baldwin I. T. (2007). Jasmonates and its mimics differentially elicit systemic defence responses in *Nicotiana attenuata*. J. Exp. Bot. 58, 4071–4082. 10.1093/jxb/erm263 18065767

[B70] Prelich G. (2012). Gene overexpression: uses, mechanisms, and interpretation. Genetics 190, 841–854. 10.1534/genetics.111.136911 22419077 PMC3296252

[B71] Rodrigues S. M. Andrade M. O. Gomes A. P. Damatta F. M. Baracat-Pereira M. C. Fontes E. P. (2006). Arabidopsis and tobacco plants ectopically expressing the soybean antiquitin-like *ALDH7* gene display enhanced tolerance to drought, salinity, and oxidative stress. J. Exp. Bot. 57, 1909–1918. 10.1093/jxb/erj132 16595581

[B72] Sánchez-Hernandez C. Martinez-Gallardo N. Guerrero-Rangel A. Valdes-Rodriguez S. Delano-Frier J. (2004). Trypsin and alpha-amylase inhibitors are differentially induced in leaves of amaranth (*Amaranthus hypochondriacus*) in response to biotic and abiotic stress. Physiol Plant. 122, 254–264. 10.1111/j.0031-9317.2004.00398.x

[B73] Schittko U. Baldwin I. T. (2003). Constraints to herbivore-induced systemic responses: bidirectional signaling along orthostichies in *Nicotiana attenuata*. J. Chem. Ecol. 29, 763–770. 10.1023/A:1022833022672 12757332

[B74] Schommer C. Palatnik J. F. Aggarwal P. Chételat A. Cubas P. Farmer E. E.. (2008). Control of jasmonate biosynthesis and senescence by miR319 targets. PLoS Biol. 6:e230. 10.1371/journal.pbio.0060230 18816164 PMC2553836

[B75] Schwechheimer C. Willige B. C. Zourelidou M. Dohmann E. M. N. (2009). Examining protein stability and its relevance for plant growth and development. Methods Mol. Biol. 479, 147–171. 10.1007/978-1-59745-289-2_10 19083189

[B76] Siré C. Moreno A. B. Garcia-Chapa M. López-Moya J. J. San Segundo B. (2009). Diurnal oscillation in the accumulation of Arabidopsis microRNAs, miR167, miR168, miR171 and miR398. FEBS Lett. 583, 1039–1044. 10.1016/j.febslet.2009.02.024 19236868

[B77] Soltis P. S. Soltis D. E. Chase M. W. (1999). Angiosperm phylogeny inferred from multiple genes as a tool for comparative biology. Nature 402, 402–404. 10.1038/46528 10586878

[B78] Stallknecht G. F. Schulz-Schaeffer J. R. (1993). Amaranth rediscovered, in New Crops, eds Janick J. Simon J. E. (New York, NY: Wiley), 211–218.

[B79] Steppuhn A. Baldwin I. T. (2007). Resistance management in a native plant: nicotine prevents herbivores from compensating for plant protease inhibitors. Ecol. Lett. 10, 499–511. 10.1111/j.1461-0248.2007.01045.x 17498149

[B80] Steppuhn A. Gase K. Kroch B. Halitschke R. Baldwin I. T. (2004). Nicotine's defensive function in nature. PLoS Biol. 2:E217. 10.1371/journal.pbio.0020217 15314646 PMC509292

[B81] Stoppin V. Vantard M. Schmit A. C. Lambert A. M. (1994). Isolated plant nuclei nucleate microtubule assembly-the nuclear surface in higher-plants has centrosome-like activity. Plant Cell 6, 1099–1106. 10.1105/tpc.6.8.1099 12244268 PMC160504

[B82] Takada S. Iida H. (2014). Specification of epidermal cell fate in plant shoots. Front. Plant Sci. 5:49. 10.3389/fpls.2014.00049 24616724 PMC3934432

[B83] Tamura K. Peterson D. Peterson N. Stecher G. Nei M. Kumar S. (2011). MEGA5: molecular evolutionary genetics analysis using maximum likelihood, evolutionary distance, and maximum parsimony methods. Mol. Biol. Evol. 28, 2731–2739. 10.1093/molbev/msr121 21546353 PMC3203626

[B84] Tanveer A. Khaliq A. Siddiqui M. H. (2013). A review on genus *Alternanthera* weeds implications. Pak. J. Weed Sci. Res. 19, 53–58.

[B85] Turner M. Nizampatnam N. R. Baron M. Coppin S. Damodaran S. Adhikari S.. (2013). Ectopic expression of miR160 results in auxin hypersensitivity, cytokinin hyposensitivity, and inhibition of symbiotic nodule development in soybean. Plant Physiol. 162, 2042–2055. 10.1104/pp.113.220699 23796794 PMC3729781

[B86] Valenzuela-Soto J. H. Iruegas-Bocardo F. Martínez-Gallardo N. A. Molina-Torres J. Gómez-Lim M. A. Délano-Frier J. P. (2011). Transformed tobacco (*Nicotiana tabacum*) plants over-expressing a peroxisome proliferator-activated receptor gene from *Xenopus laevis* (xPPARa) show increased susceptibility to infection by virulent *Pseudomonas syringae* pathogens. Planta 233, 507–521. 10.1007/s00425-010-1314-7 21104271

[B88] Vargas-Ortiz E. Délano-Frier J. P. Tiessen A. (2015). The tolerance of grain amaranth (*Amaranthus cruentus* L.) to defoliation during vegetative growth is compromised during flowering. Plant Physiol. Biochem. 91, 36–40. 10.1016/j.plaphy.2015.03.007 25863889

[B87] Vargas-Ortiz E. Espitia-Rangel E. Tiessen A. Délano-Frier J. P. (2013). Grain amaranths are defoliation tolerant crop species capable of utilizing stem and root carbohydrate reserves to sustain vegetative and reproductive growth after leaf loss. PLoS ONE 8:e67879. 10.1371/journal.pone.0067879 23861825 PMC3701626

[B89] Venskutonis P. R. Kraujalis P. (2013). Nutritional components of amaranth seeds and vegetables: a review on composition, properties, and uses. Compr. Rev. Food Sci. Food Saf. 12, 381–412. 10.1111/1541-4337.12021 33412681

[B90] Venu R. C. Sreereka M. V. Sheshu-Madhav M. Nobuta K. Madhan-Mohan K. Chen S. (2013). Deep transcriptome sequencing reveals the expression of key functional and regulatory genes involved in the abiotic stress signaling pathways in rice. J. Plant Biol. 56, 216–231. 10.1007/s12374-013-0075-9

[B91] Voelker T. Sturm A. Chrispeels M. J. (1987). Differences in expression between two seed lectin alleles obtained from normal and lectin-deficient beans are maintained in transgenic tobacco. EMBO J. 6, 3571–3577. 16453809 10.1002/j.1460-2075.1987.tb02687.x PMC553823

[B92] Watson J. M. Riha K. (2010). Comparative biology of telomeres: where plants stand. FEBS Lett. 584, 3752–3759. 10.1016/j.febslet.2010.06.017 20580356 PMC3767043

[B93] Yi X. Du Z. Su Z. (2013). PlantGSEA: a gene set enrichment analysis toolkit for plant community. Nucleic Acids Res. 41, W98–W103. 10.1093/nar/gkt281 23632162 PMC3692080

[B94] Ying S. Zhang D. F. Fu J. Shi Y. S. Song Y. C. Wang T. Y.. (2012). Cloning and characterization of a maize bZIP transcription factor, ZmbZIP72, confers drought and salt tolerance in transgenic *Arabidopsis*. Planta 235, 253–266. 10.1007/s00425-011-1496-7 21866346

[B95] Zavala J. A. Patankar A. G. Gase K. Hui D. Baldwin I. T. (2004). Manipulation of endogenous trypsin proteinase inhibitor production in *Nicotiana attenuata* demonstrates their function as antiherbivore defenses. Plant Physiol. 134, 1181–1190. 10.1104/pp.103.035634 14976235 PMC389942

[B96] Zhang Y. Zhang B. Yan D. Dong W. Yang W. Li Q.. (2011). Two Arabidopsis cytochrome P450 monooxygenases, CYP714A1 and CYP714A2, function redundantly in plant development through gibberellin deactivation. Plant J. 67, 342–353. 10.1111/j.1365-313X.2011.04596.x 21457373

[B97] Zhou D. X. (1999). Regulatory mechanism of plant gene transcription by GT-elements and GT-factors. Trends Plant Sci. 4, 210–214. 10.1016/S1360-1385(99)01418-1 10366876

[B98] Zhou J. Wang B. Li Y. Wang Y. Zhu L. (2007). Responses of chrysanthemum cells to mechanical stimulation require intact microtubules and plasma membrane-cell wall adhesion. J. Plant Growth Regul. 26, 55–68. 10.1007/s00344-006-0029-2

[B99] Zhu C. Ding Y. Liu H. (2011). MiR398 and plant stress responses. Physiol. Plant. 143, 1–9. 10.1111/j.1399-3054.2011.01477.x 21496029

[B100] Zhu Y. Nomura T. Xu Y. Zhang Y. Peng Y. Mao B.. (2006). *ELONGATED UPPERMOST INTERNODE* encodes a cytochrome P450 monooxygenase that epoxidizes gibberellins in a novel deactivation reaction in rice. Plant Cell 18, 442–456. 10.1105/tpc.105.038455 16399803 PMC1356550

