# Supplementary figures and images for: The novel and taxonomically restricted Ah24 gene from grain amaranth (Amaranthus hypochondriacus) has a dual role in development and defense
Source: Front Plant Sci. 2015 Aug 5;6:602. doi: 10.3389/fpls.2015.00602 (PMC4524895; doi:10.3389/fpls.2015.00602)

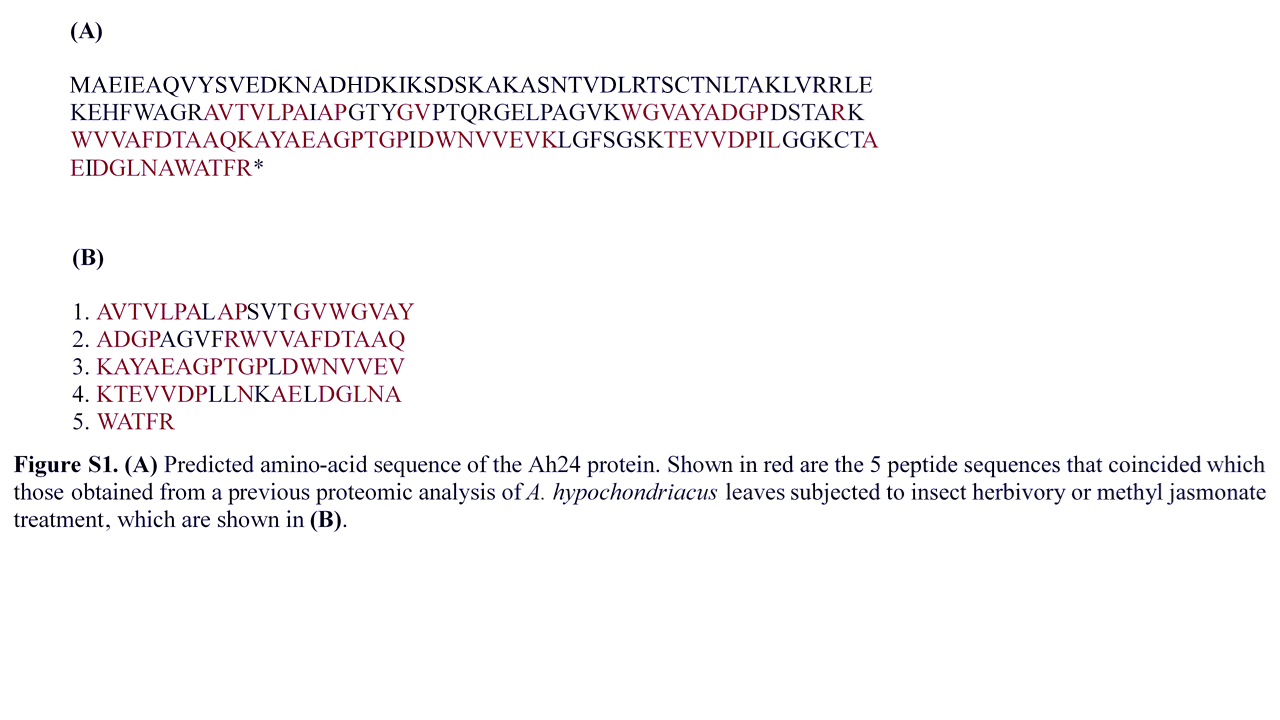

Supplement: Supplementary file 1 [file Image1.TIF]

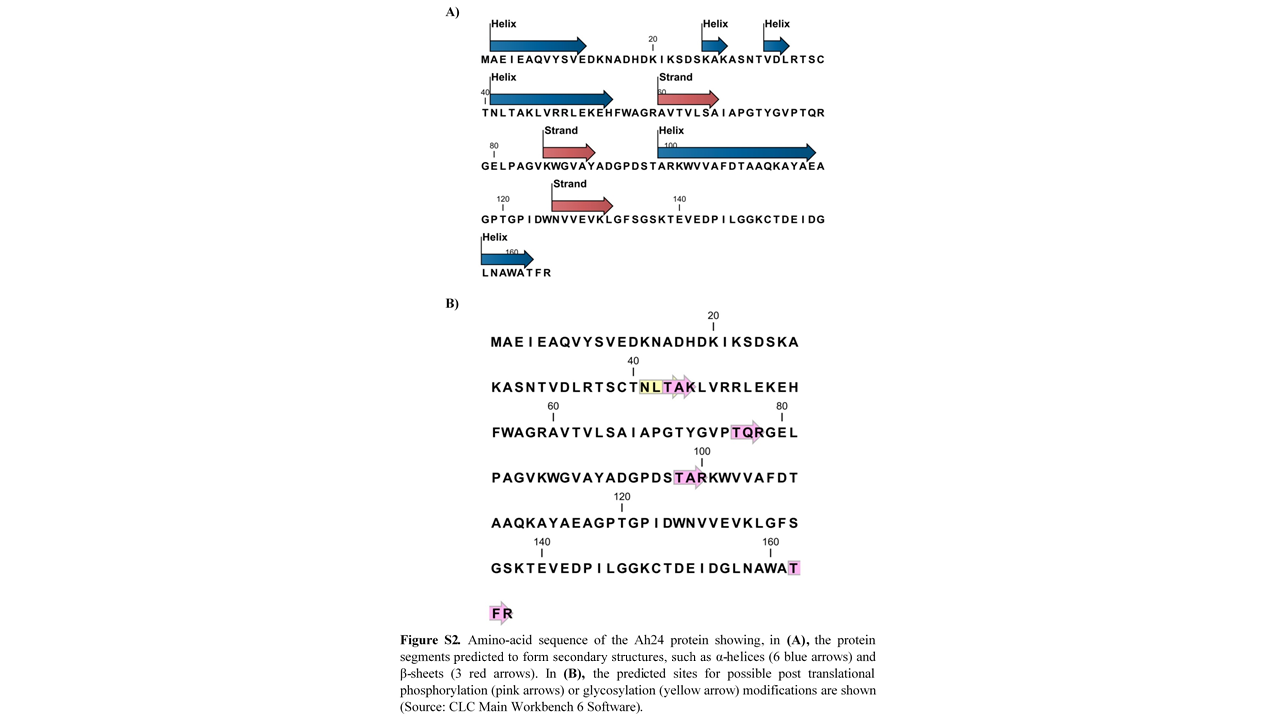

Supplement: Supplementary file 2 [file Image2.TIF]

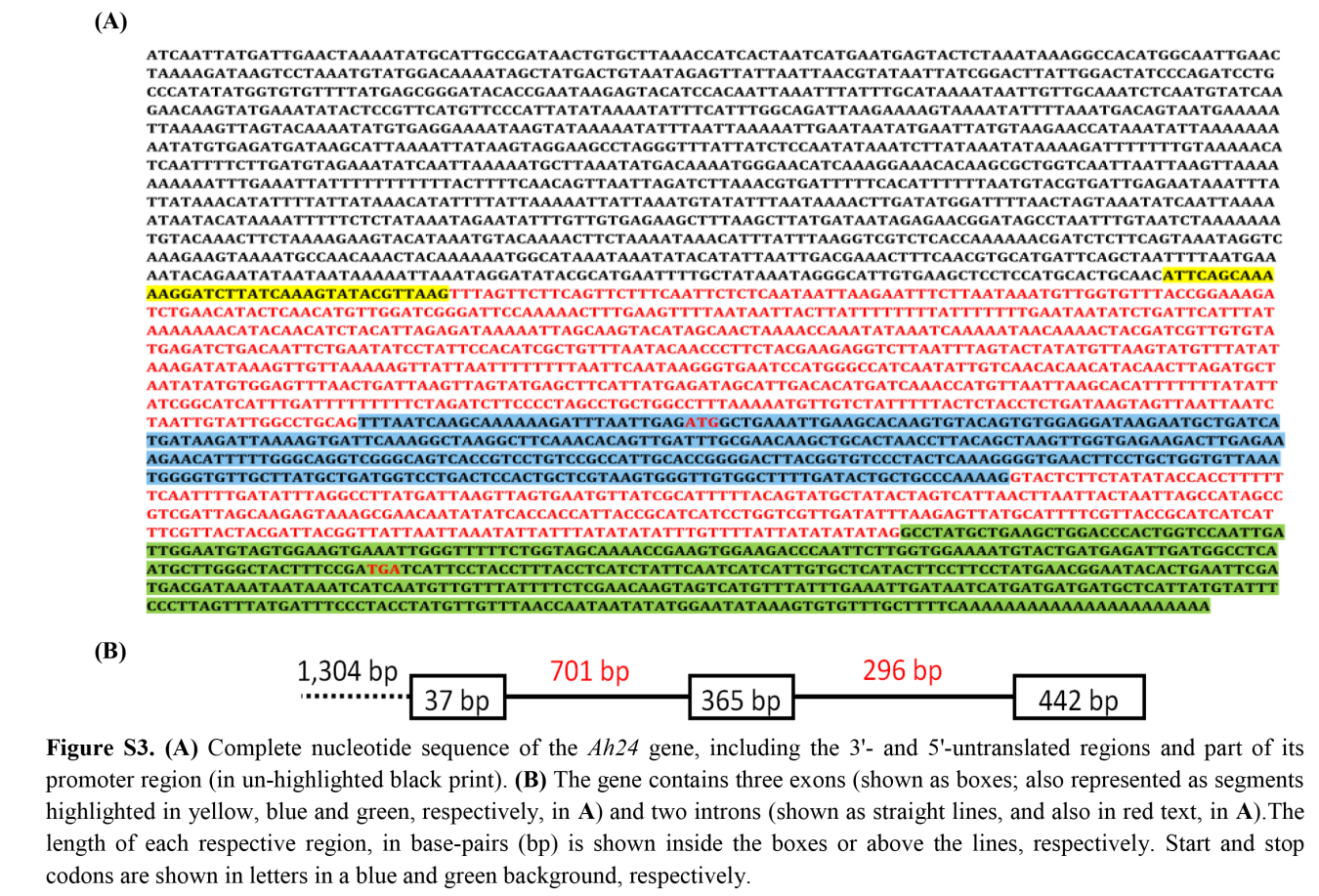

Supplement: Supplementary file 3 [file Image3.TIF]

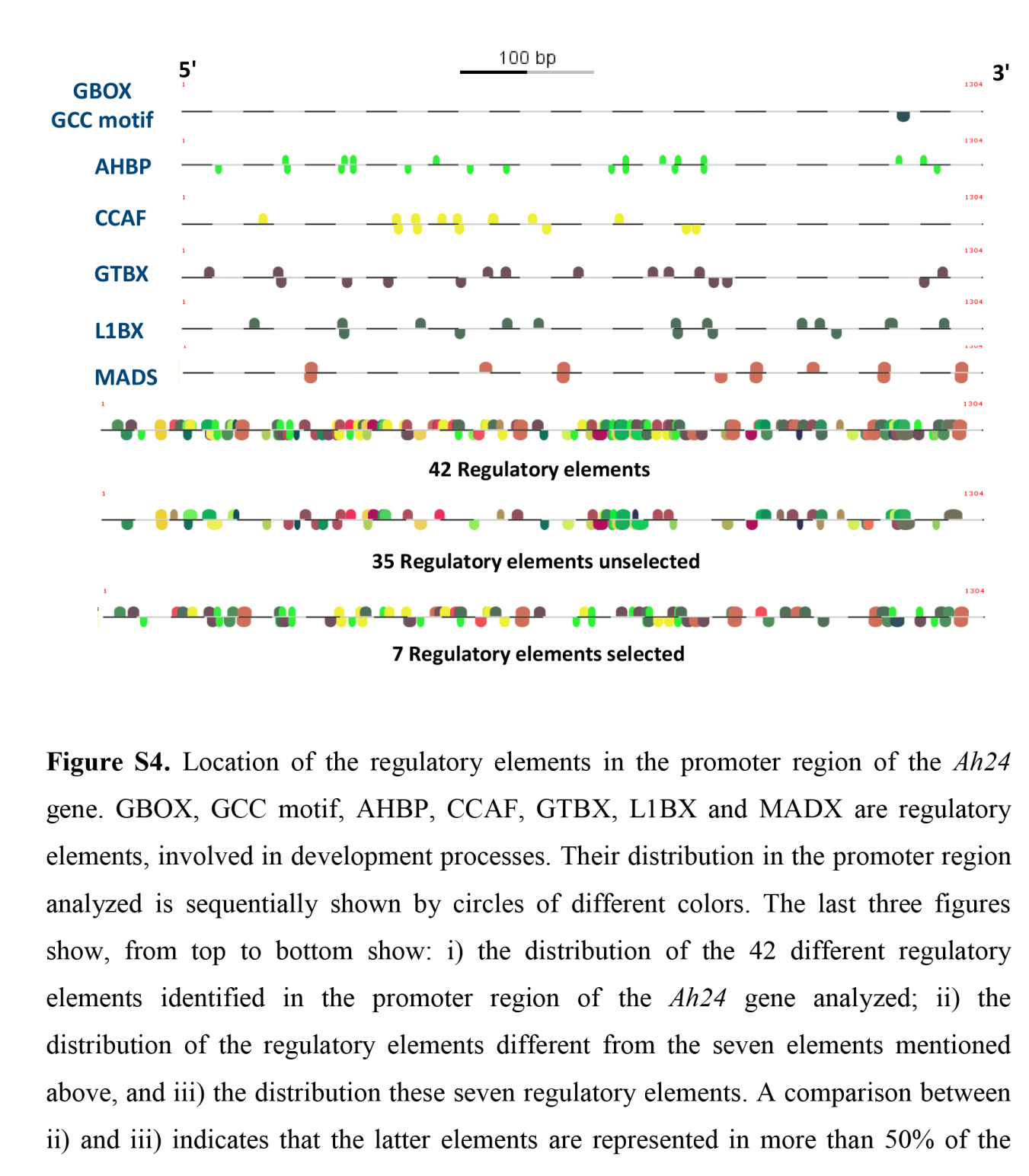

Supplement: Supplementary file 4 [file Image4.TIF]

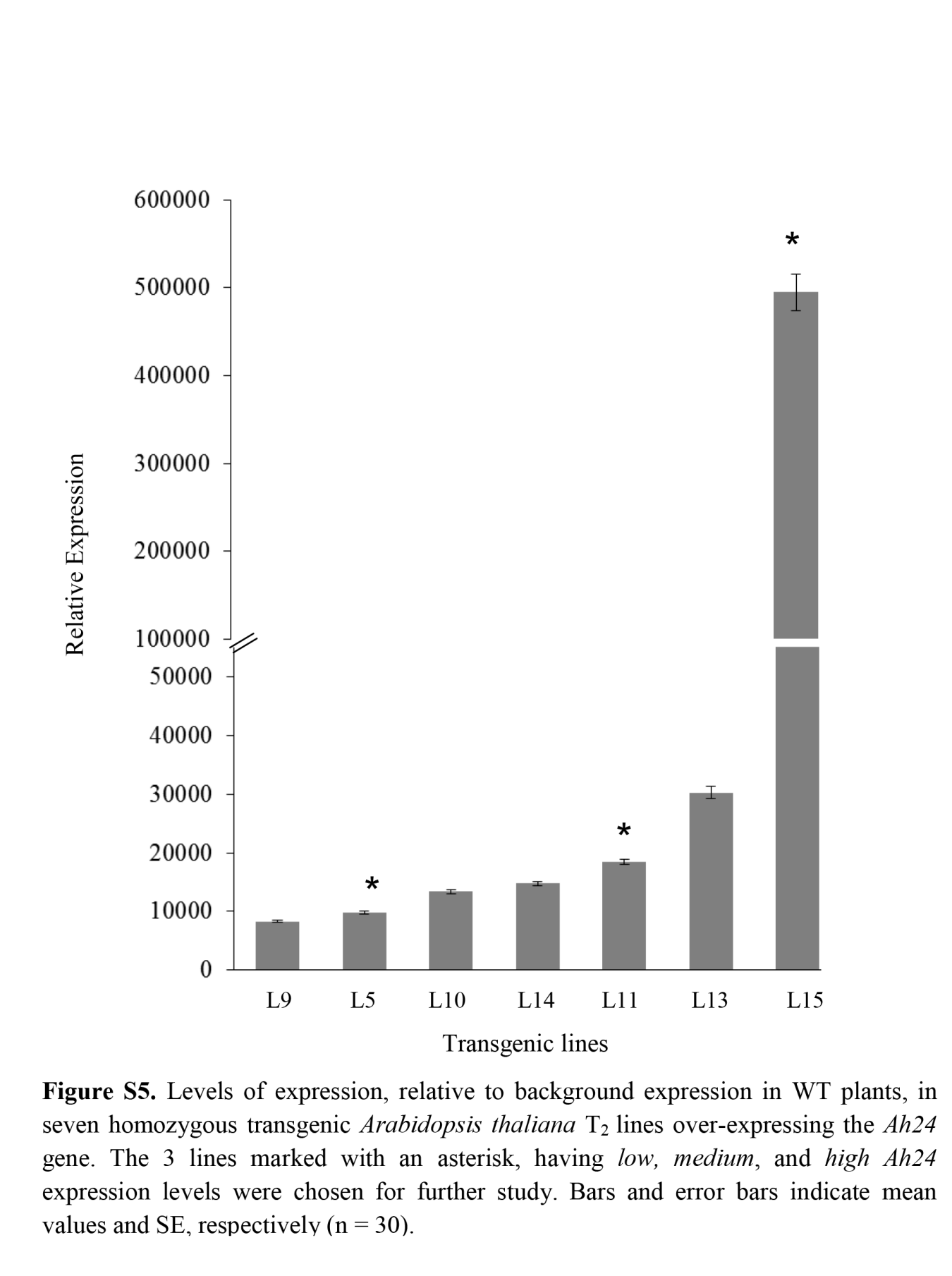

Supplement: Supplementary file 5 [file Image5.TIF]

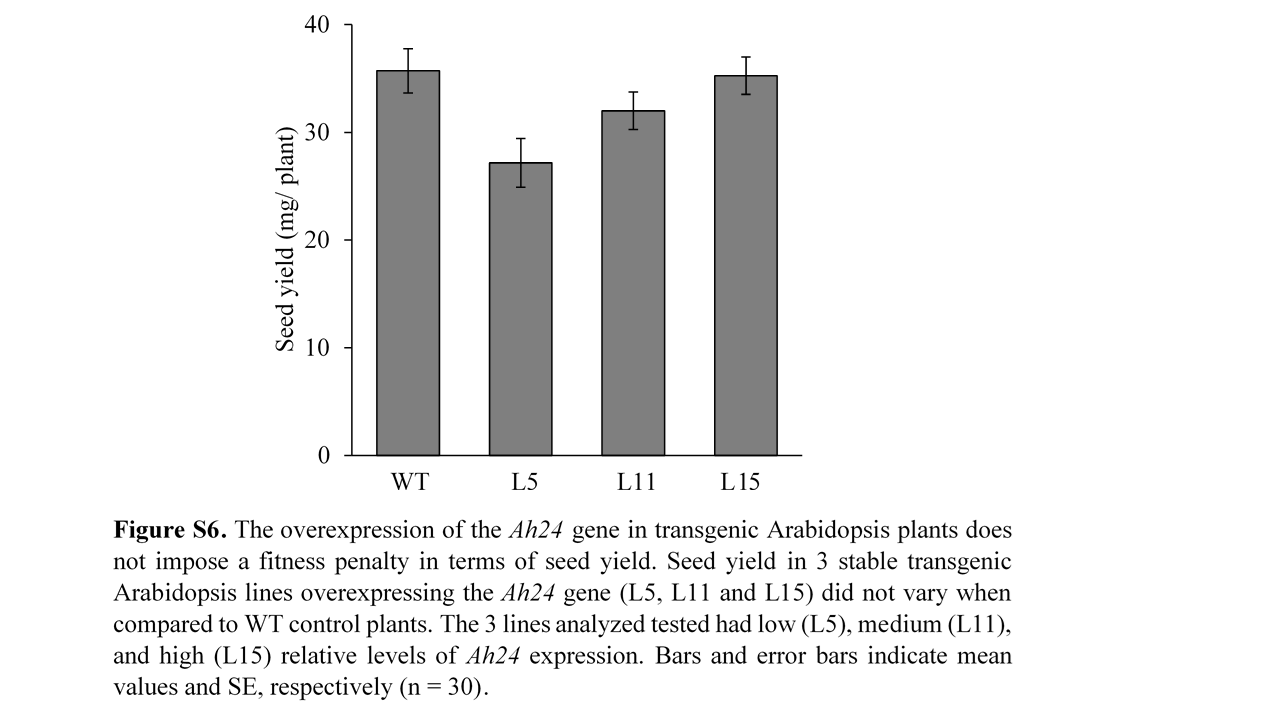

Supplement: Supplementary file 6 [file Image6.TIF]

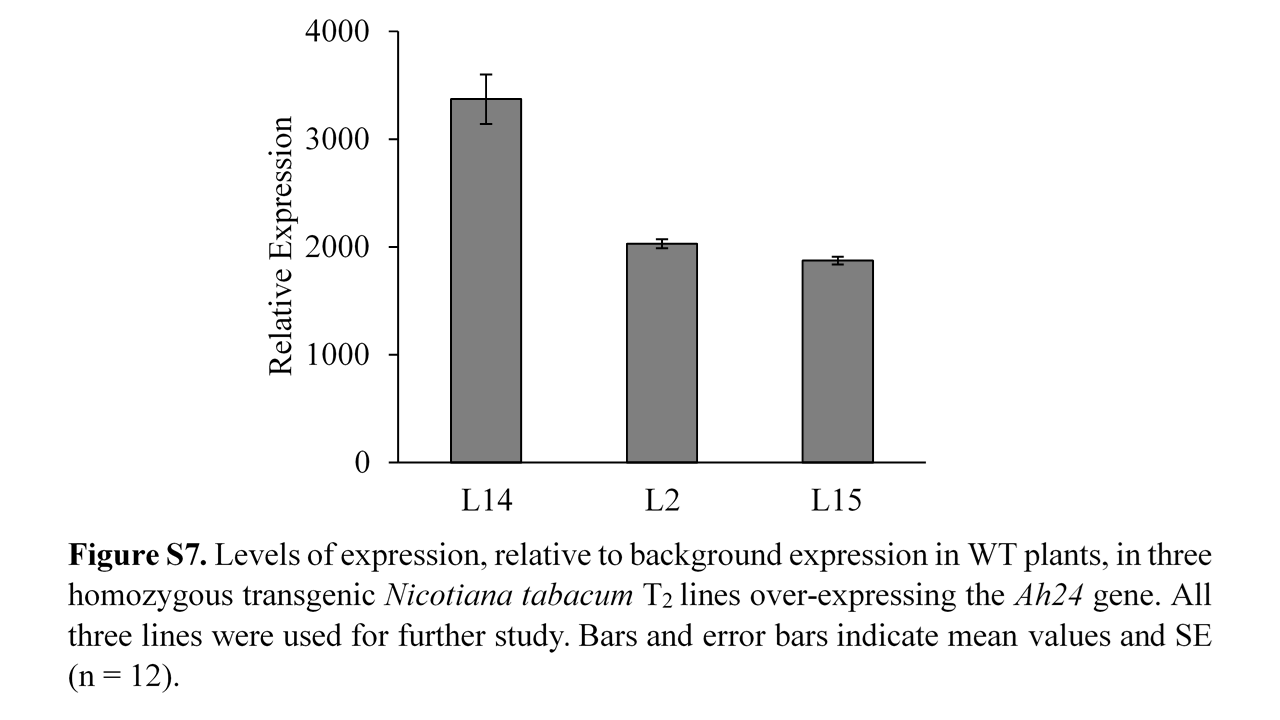

Supplement: Supplementary file 7 [file Image7.TIF]

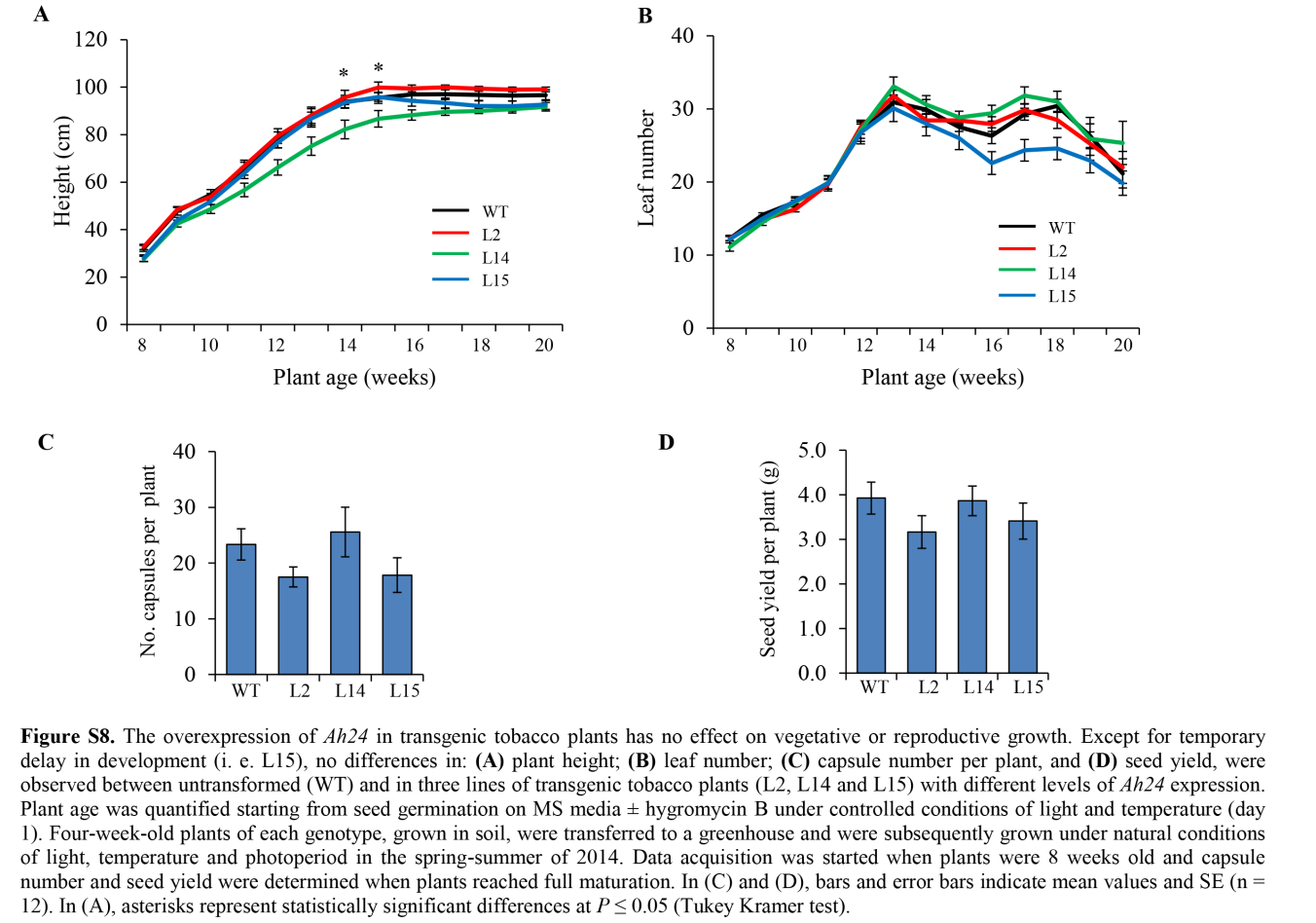

Supplement: Supplementary file 8 [file Image8.TIF]

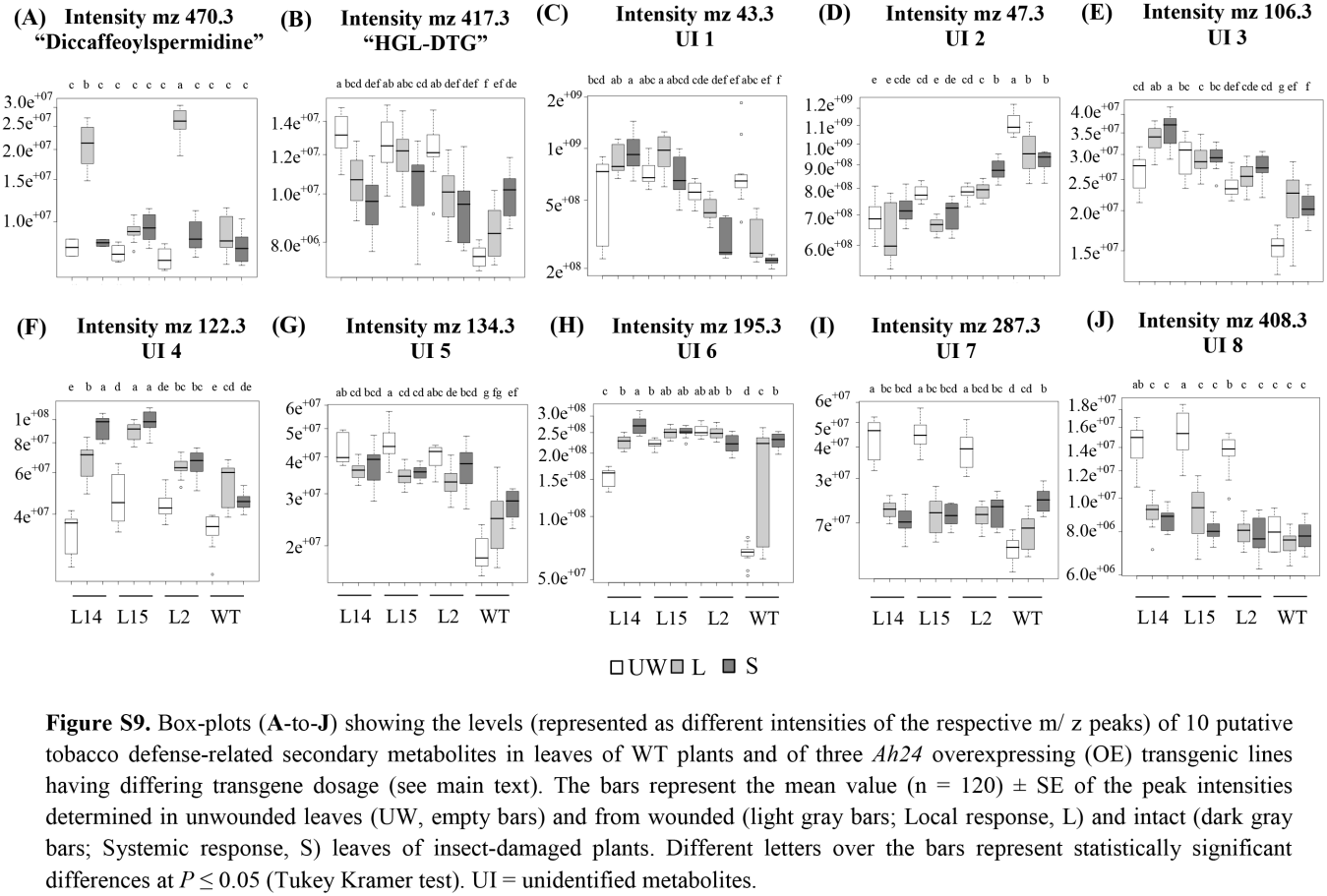

Supplement: Supplementary file 9 [file Image9.TIF]

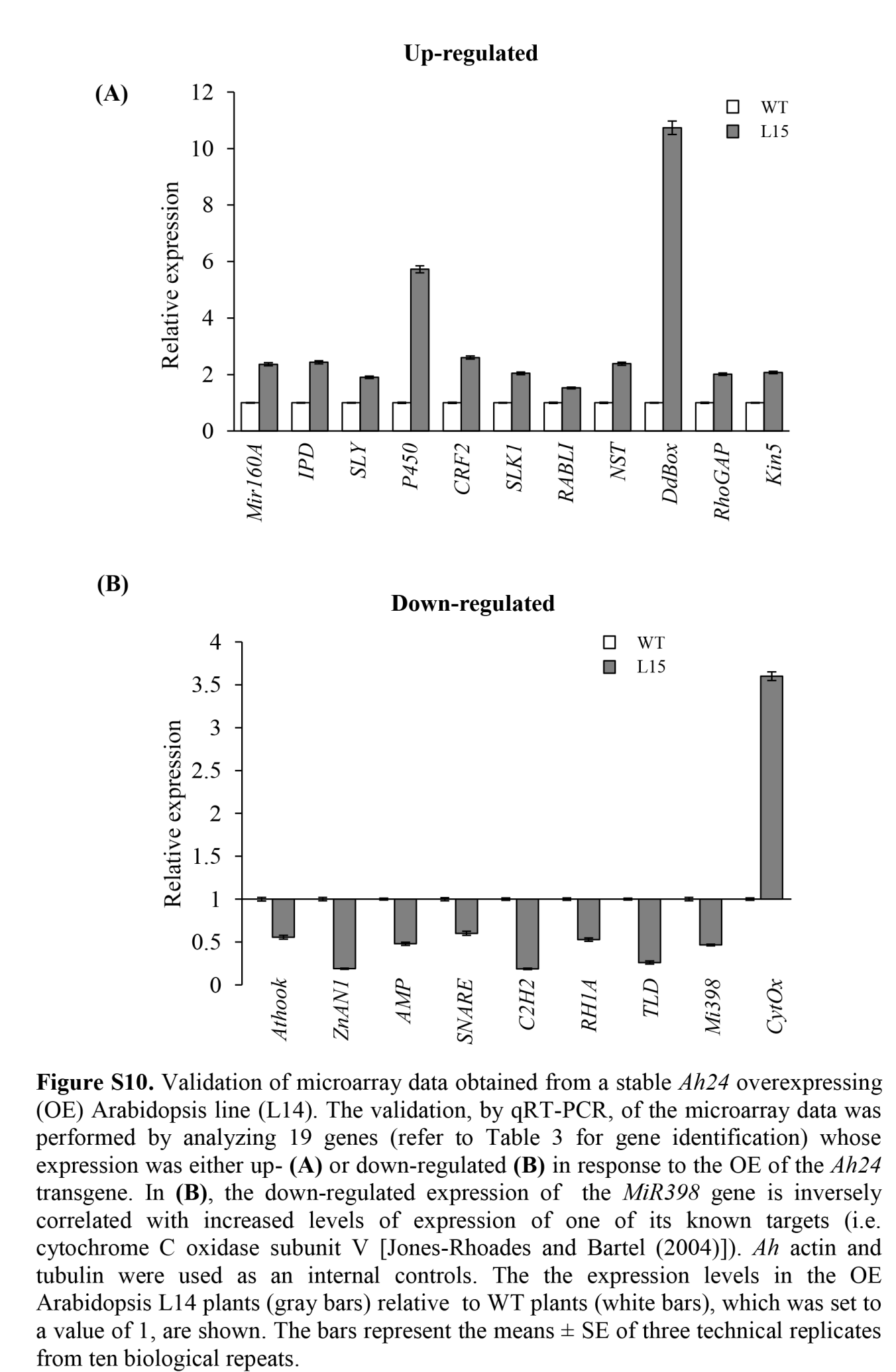

Supplement: Supplementary file 10 [file Image10.TIF]

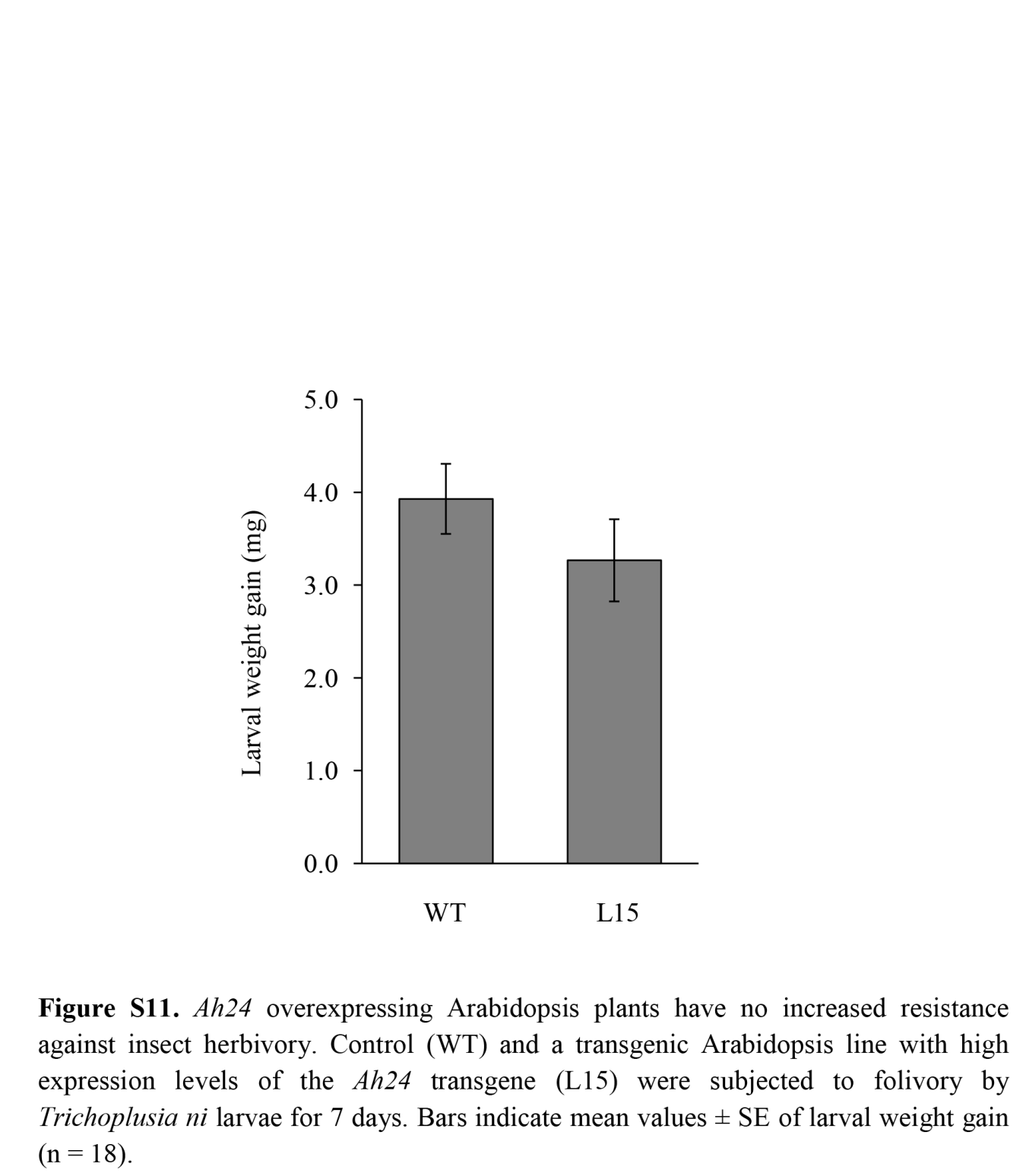

Supplement: Supplementary file 11 [file Image11.TIF]
