# Supplementary material for: The novel and taxonomically restricted Ah24 gene from grain amaranth (Amaranthus hypochondriacus) has a dual role in development and defense
Source: Front Plant Sci. 2015 Aug 5;6:602. doi: 10.3389/fpls.2015.00602 (PMC4524895; doi:10.3389/fpls.2015.00602)
Supplement: Supplementary file 12 [file Table1.DOCX]

**Table S1.** Cis-regulatory elements found in the promoter region of the *Ah24* gene. Additional columns indicate the distance from the start codon of each element instance present in the Ah24 promoter and their consensus nucleotide sequences of the DNA binding sites.

| **Motif type** | **Detailed family information** | \| **Localization** \| \| --- \| | | | **Matrix** | **Sequence** |
| --- | --- | --- | --- | --- | --- | --- | --- |
|  |  | **from** | **to** | **strand** |  |  |
| ABRE | ABA response elements | 81 | 97 | (+) | 0.889 | aaaggCCACatggcaat |
| ABRE | ABA response elements | 82 | 98 | (-) | 0.882 | aattgCCATgtggcctt |
| AGP1 | Plant GATA-type zinc finger protein | 751 | 761 | (-) | 0.988 | taaGATCtaat |
| AGP1 | Plant GATA-type zinc finger protein | 752 | 762 | (+) | 0.926 | ttaGATCttaa |
| AHBP | Arabidopsis homeobox protein | 803 | 813 | (+) | 0.936 | aaaTTTAttat |
| AHBP | Arabidopsis homeobox protein | 798 | 808 | (-) | 0.936 | aaaTTTAttct |
| AHBP | Arabidopsis homeobox protein | 778 | 788 | (+) | 1 | tttttTAATgt |
| AHBP | Arabidopsis homeobox protein | 1162 | 1172 | (+) | 0.931 | catATTAattg |
| AHBP | Arabidopsis homeobox protein | 845 | 855 | (+) | 0.998 | aaaATTAttaa |
| AHBP | Arabidopsis homeobox protein | 845 | 855 | (-) | 0.903 | ttaATAAtttt |
| AHBP | Arabidopsis homeobox protein | 165 | 175 | (+) | 0.914 | cgtataATTAt |
| AHBP | Arabidopsis homeobox protein | 168 | 178 | (-) | 0.929 | ccgataATTAt |
| AHBP | Arabidopsis homeobox protein | 718 | 728 | (-) | 0.901 | aaaATAAtttc |
| AHBP | Arabidopsis homeobox protein | 718 | 728 | (+) | 0.998 | gaaATTAtttt |
| AHBP | Arabidopsis homeobox protein | 695 | 705 | (-) | 0.925 | taaCTTAatta |
| AHBP | Arabidopsis homeobox protein | 524 | 534 | (-) | 1 | aatttTAATgc |
| AHBP | Arabidopsis homeobox protein | 465 | 475 | (-) | 0.929 | catATTAttca |
| AHBP | Arabidopsis homeobox protein | 410 | 420 | (+) | 0.92 | aaaGTTAgtac |
| AHBP | Arabidopsis homeobox protein | 1202 | 1212 | (+) | 1 | aatttTAATga |
| AHBP | Arabidopsis homeobox protein | 256 | 266 | (-) | 0.938 | aaaTTTAattg |
| AHBP | Arabidopsis homeobox protein | 1224 | 1234 | (-) | 0.923 | tttattATTAt |
| AHBP | Arabidopsis homeobox protein | 261 | 271 | (+) | 0.936 | aaaTTTAtttg |
| AHBP | Arabidopsis homeobox protein | 364 | 374 | (-) | 0.963 | tttctTAATct |
| AHBP | Arabidopsis homeobox protein | 275 | 285 | (+) | 0.901 | aaaATAAttgt |
| AHBP | Arabidopsis homeobox protein | 275 | 285 | (-) | 1 | acaATTAtttt |
| AHBP | Arabidopsis homeobox protein | 56 | 66 | (-) | 0.907 | ttcatgATTAg |
| ASRC | AS1/AS2 repressor complex | 1180 | 1188 | (-) | 0.867 | acgTTGAaa |
| CAAT | CCAAT binding factors | 562 | 570 | (+) | 0.98 | ctCCAAtat |
| CAAT | CCAAT binding factors | 181 | 189 | (-) | 0.97 | gtCCAAtaa |
| CARM | CA-rich motif | 736 | 754 | (+) | 0.791 | acttttcAACAgttaatta |
| CCAF | Circadian control factors | 343 | 357 | (+) | 0.897 | atataaAATAtttca |
| CCAF | Circadian control factors | 346 | 360 | (-) | 0.866 | aaatgaAATAtttta |
| CCAF | Circadian control factors | 587 | 601 | (-) | 0.949 | tacaaaaaAATCttt |
| CCAF | Circadian control factors | 564 | 578 | (+) | 0.872 | ccaatataAATCtta |
| CCAF | Circadian control factors | 374 | 388 | (+) | 0.899 | aagtaaAATAtttta |
| CCAF | Circadian control factors | 377 | 391 | (-) | 0.899 | atttaaAATAtttta |
| CCAF | Circadian control factors | 126 | 140 | (+) | 0.862 | ggacaaAATAgctat |
| CCAF | Circadian control factors | 501 | 515 | (+) | 0.9 | aaaaaaAATAtgtga |
| CCAF | Circadian control factors | 814 | 828 | (-) | 0.894 | taataaAATAtgttt |
| CCAF | Circadian control factors | 705 | 719 | (+) | 0.872 | aaaaaaaaAATTtga |
| CCAF | Circadian control factors | 830 | 844 | (-) | 0.894 | taataaAATAtgttt |
| CCAF | Circadian control factors | 445 | 459 | (-) | 0.865 | taattaAATAttttt |
| CCAF | Circadian control factors | 442 | 456 | (+) | 0.901 | tataaaAATAtttaa |
| CCAF | Circadian control factors | 417 | 431 | (+) | 0.895 | gtacaaAATAtgtga |
| CCAF | Circadian control factors | 499 | 513 | (+) | 0.85 | taaaaaaaAATAtgt |
| DOFF | DNA binding with one finger (DOF) | 727 | 743 | (-) | 0.768 | tgaaaagtAAAAaaaaa |
| DOFF | DNA binding with one finger (DOF) | 395 | 411 | (+) | 0.776 | agtaatgaAAAAttaaa |
| DOFF | DNA binding with one finger (DOF) | 732 | 748 | (-) | 0.788 | actgttgaAAAGtaaaa |
| DOFF | DNA binding with one finger (DOF) | 953 | 969 | (-) | 0.986 | ataagcttAAAGcttct |
| EINL | Ethylen insensitive 3 like factors | 785 | 793 | (+) | 0.941 | aTGTAcgtg |
| EINL | Ethylen insensitive 3 like factors | 1028 | 1036 | (-) | 0.935 | aTGTActtc |
| EREF | Ethylen respone element factors | 1172 | 1190 | (+) | 0.909 | gACGAaactttcaacgtgc |
| GAPB | GAP-Box (light response elements) | 395 | 409 | (+) | 0.893 | agtaATGAaaaatta |
| GAPB | GAP-Box (light response elements) | 493 | 507 | (+) | 0.88 | aaatATTAaaaaaaa |
| GAPB | GAP-Box (light response elements) | 362 | 376 | (+) | 0.887 | gcagATTAagaaaag |
| GARP | Myb-related DNA binding proteins (Golden2, ARR, Psr) | 196 | 204 | (+) | 0.973 | AGATcctgc |
| GARP | Myb-related DNA binding proteins (Golden2, ARR, Psr) | 286 | 294 | (-) | 0.972 | AGATttgca |
| GBOX | Plant G-box/C-box bZIP proteins | 1164 | 1184 | (-) | 0.84 | tgaaagttTCGTcaattaata |
| GTBX | GT-box elements | 262 | 278 | (-) | 0.805 | ttttATGCaaataaatt |
| GTBX | GT-box elements | 638 | 654 | (+) | 0.875 | aaaatgcTTAAatatga |
| GTBX | GT-box elements | 759 | 775 | (+) | 0.994 | ttaaacGTGAtttttca |
| GTBX | GT-box elements | 858 | 874 | (-) | 0.886 | agttttaTTAAatatac |
| GTBX | GT-box elements | 1230 | 1246 | (+) | 0.873 | ataaaaaTTAAatagga |
| GTBX | GT-box elements | 1200 | 1216 | (-) | 0.913 | attttcaTTAAaattag |
| GTBX | GT-box elements | 520 | 536 | (+) | 0.881 | ataagcaTTAAaattat |
| GTBX | GT-box elements | 785 | 801 | (+) | 0.967 | atgtacGTGAttgagaa |
| GTBX | GT-box elements | 447 | 463 | (-) | 0.891 | tttttaaTTAAatattt |
| GTBX | GT-box elements | 835 | 851 | (+) | 0.875 | tattttaTTAAaaatta |
| GTBX | GT-box elements | 880 | 896 | (-) | 0.906 | ttactagTTAAaatcca |
| GTBX | GT-box elements | 38 | 54 | (+) | 0.881 | actgtgcTTAAaccatc |
| GTBX | GT-box elements | 150 | 166 | (+) | 0.897 | agagttaTTAAttaacg |
| GTBX | GT-box elements | 491 | 507 | (+) | 0.879 | ataaataTTAAaaaaaa |
| GTBX | GT-box elements | 155 | 171 | (-) | 0.943 | ttatacGTTAattaata |
| GTBX | GT-box elements | 329 | 345 | (-) | 0.886 | tataATGGgaacatgaa |
| HAET | Heat shock factors | 1168 | 1184 | (-) | 0.759 | tgaaagTTTCgtcaatt |
| HAET | Heat shock factors | 1043 | 1059 | (+) | 0.757 | acaaaaCTTCtaaaata |
| HAET | Heat shock factors | 294 | 310 | (+) | 0.814 | tcaatgtatcaAGAAca |
| HAET | Heat shock factors | 1013 | 1029 | (+) | 0.765 | tacaaaCTTCtaaaaga |
| HAET | Heat shock factors | 607 | 623 | (+) | 0.776 | atcaatTTTCttgatgt |
| HMGF | High mobility group factors | 342 | 356 | (-) | 0.925 | gaaaTATTttatata |
| HMGF | High mobility group factors | 815 | 829 | (+) | 0.911 | aacaTATTttattat |
| HMGF | High mobility group factors | 446 | 460 | (+) | 0.92 | aaaaTATTtaattaa |
| HMGF | High mobility group factors | 270 | 284 | (-) | 0.927 | caatTATTttatgca |
| HMGF | High mobility group factors | 831 | 845 | (+) | 0.911 | aacaTATTttattaa |
| HMGF | High mobility group factors | 719 | 733 | (+) | 0.94 | aaatTATTttttttt |
| HMGF | High mobility group factors | 347 | 361 | (+) | 0.891 | aaaaTATTtcatttg |
| IBOX | Plant I-Box sites | 551 | 567 | (-) | 0.94 | ttggaGATAataaaccc |
| IBOX | Plant I-Box sites | 514 | 530 | (+) | 0.96 | gagatGATAagcattaa |
| IBOX | Plant I-Box sites | 965 | 981 | (+) | 0.961 | cttatGATAatagagaa |
| IBOX | Plant I-Box sites | 103 | 119 | (+) | 0.986 | taaaaGATAagtcctaa |
| IDDF | ID domain factors | 649 | 661 | (-) | 0.945 | cattTTGTcatat |
| IDDF | ID domain factors | 123 | 135 | (-) | 0.98 | tattTTGTccata |
| L1BX | L1 box, motif for L1 layer-specific expression | 856 | 872 | (-) | 0.85 | ttttatTAAAtatacat |
| L1BX | L1 box, motif for L1 layer-specific expression | 1148 | 1164 | (+) | 0.825 | cataaaTAAAtatacat |
| L1BX | L1 box, motif for L1 layer-specific expression | 1144 | 1160 | (+) | 0.852 | atggcaTAAAtaaatat |
| L1BX | L1 box, motif for L1 layer-specific expression | 522 | 538 | (+) | 0.807 | aagCATTaaaattataa |
| L1BX | L1 box, motif for L1 layer-specific expression | 847 | 863 | (+) | 0.956 | aattatTAAAtgtatat |
| L1BX | L1 box, motif for L1 layer-specific expression | 796 | 812 | (+) | 0.834 | tgagaaTAAAtttatta |
| L1BX | L1 box, motif for L1 layer-specific expression | 381 | 397 | (+) | 0.879 | atatttTAAAtgacagt |
| L1BX | L1 box, motif for L1 layer-specific expression | 799 | 815 | (-) | 0.861 | ttataaTAAAtttattc |
| L1BX | L1 box, motif for L1 layer-specific expression | 573 | 589 | (+) | 0.831 | atcttaTAAAtataaaa |
| L1BX | L1 box, motif for L1 layer-specific expression | 1232 | 1248 | (+) | 0.82 | aaaaatTAAAtaggata |
| L1BX | L1 box, motif for L1 layer-specific expression | 445 | 461 | (-) | 0.783 | tttaatTAAAtattttt |
| L1BX | L1 box, motif for L1 layer-specific expression | 1001 | 1017 | (+) | 0.821 | tctaaaAAAAtgtacaa |
| L1BX | L1 box, motif for L1 layer-specific expression | 254 | 270 | (+) | 0.791 | cacaatTAAAtttattt |
| L1BX | L1 box, motif for L1 layer-specific expression | 257 | 273 | (-) | 0.821 | tgcaaaTAAAtttaatt |
| L1BX | L1 box, motif for L1 layer-specific expression | 1057 | 1073 | (-) | 0.874 | cttaaaTAAAtgtttat |
| L1BX | L1 box, motif for L1 layer-specific expression | 111 | 127 | (+) | 0.917 | aagtccTAAAtgtatgg |
| L1BX | L1 box, motif for L1 layer-specific expression | 1030 | 1046 | (+) | 0.924 | agtacaTAAAtgtacaa |
| LEGB | Legumin Box family | 1175 | 1201 | (-) | 0.897 | agctgaatCATGcacgttgaaagtttc |
| LREM | Light responsive element motif, not modulated by different light qualities | 998 | 1008 | (+) | 0.98 | taATCTaaaaa |
| LREM | Light responsive element motif, not modulated by different light qualities | 749 | 759 | (-) | 0.921 | agATCTaatta |
| MADS | MADS box proteins | 1133 | 1153 | (+) | 0.927 | aactacaaaAAATggcataaa |
| MADS | MADS box proteins | 199 | 219 | (-) | 0.883 | acacaCCATatatgggcagga |
| MADS | MADS box proteins | 867 | 887 | (-) | 0.865 | aaaatCCATatcaagttttat |
| MADS | MADS box proteins | 611 | 631 | (+) | 0.864 | attttcttgatgtaGAAAtat |
| MADS | MADS box proteins | 1258 | 1278 | (-) | 0.872 | atgccCTATttatagcaaaat |
| MADS | MADS box proteins | 1017 | 1037 | (+) | 0.84 | aacTTCTaaaagaagtacata |
| MADS | MADS box proteins | 925 | 945 | (+) | 0.968 | tttctctatAAATagaatatt |
| MADS | MADS box proteins | 610 | 630 | (-) | 0.868 | tatttctacatcaaGAAAatt |
| MADS | MADS box proteins | 484 | 504 | (+) | 0.908 | aagaaccatAAATattaaaaa |
| MADS | MADS box proteins | 1259 | 1279 | (+) | 0.968 | ttttgctatAAATagggcatt |
| MADS | MADS box proteins | 200 | 220 | (+) | 0.883 | cctgcCCATatatggtgtgtt |
| MADS | MADS box proteins | 1132 | 1152 | (-) | 0.881 | ttatgCCATtttttgtagttt |
| MADS | MADS box proteins | 924 | 944 | (-) | 0.881 | atattCTATttatagagaaaa |
| MIIG | MYB IIG-type binding sites | 1126 | 1140 | (-) | 0.91 | ttgtagtttgTTGGc |
| MSAE | M-phase-specific activator elements | 321 | 335 | (-) | 0.813 | acatgAACGgagtat |
| MSAE | M-phase-specific activator elements | 975 | 989 | (+) | 0.835 | tagagAACGgatagc |
| MYBL | MYB-like proteins | 409 | 425 | (+) | 0.792 | aaaagtTAGTacaaaat |
| MYBL | MYB-like proteins | 31 | 47 | (-) | 0.921 | taagcacaGTTAtcggc |
| MYBL | MYB-like proteins | 1056 | 1072 | (+) | 0.806 | aataaacaTTTAtttaa |
| MYBL | MYB-like proteins | 1123 | 1139 | (-) | 0.859 | tgtagtTTGTtggcatt |
| MYBL | MYB-like proteins | 739 | 755 | (+) | 0.884 | tttcaaCAGTtaattag |
| MYBS | MYB proteins with single DNA binding repeat | 1152 | 1168 | (-) | 0.807 | taatatgtaTATTtatt |
| MYBS | MYB proteins with single DNA binding repeat | 1242 | 1258 | (+) | 0.936 | taggATATacgcatgaa |
| MYBS | MYB proteins with single DNA binding repeat | 180 | 196 | (-) | 0.831 | tgggATAGtccaataag |
| MYBS | MYB proteins with single DNA binding repeat | 1237 | 1253 | (-) | 0.976 | gcgtatATCCtatttaa |
| MYBS | MYB proteins with single DNA binding repeat | 976 | 992 | (-) | 0.936 | taggctATCCgttctct |
| MYBS | MYB proteins with single DNA binding repeat | 922 | 938 | (+) | 0.858 | atttttctcTATAaata |
| MYBS | MYB proteins with single DNA binding repeat | 18 | 34 | (+) | 0.848 | taaaATATgcattgccg |
| MYBS | MYB proteins with single DNA binding repeat | 185 | 201 | (+) | 0.901 | tggactATCCcagatcc |
| NACF | Plant specific NAC [NAM (no apical meristem), ATAF172, CUC2 (cup-shaped cotyledons 2)] transcription factors | 1181 | 1193 | (-) | 0.961 | catgCACGttgaa |
| NCS1 | Nodulin consensus sequence 1 | 103 | 113 | (+) | 0.951 | tAAAAgataag |
| NCS1 | Nodulin consensus sequence 1 | 585 | 595 | (+) | 0.953 | tAAAAgatttt |
| NCS1 | Nodulin consensus sequence 1 | 1057 | 1067 | (-) | 0.857 | tAAATgtttat |
| NCS1 | Nodulin consensus sequence 1 | 1087 | 1097 | (+) | 0.858 | aAAACgatctc |
| OCSE | Enhancer element first identified in the promoter of the octopine synthase gene (OCS) of the Agrobacterium tumefaciens T-DNA | 150 | 170 | (+) | 0.734 | agagttattaattaACGTata |
| OCSE | Enhancer element first identified in the promoter of the octopine synthase gene (OCS) of the Agrobacterium tumefaciens T-DNA | 1159 | 1179 | (+) | 0.768 | atacatattaattGACGaaac |
|  |  |  |  |  |  |  |
| OCSE | Enhancer element first identified in the promoter of the octopine synthase gene (OCS) of the Agrobacterium tumefaciens T-DNA | 760 | 780 | (-) | 0.798 | aaatgtgaaaaatcACGTtta |
| OCSE | Enhancer element first identified in the promoter of the octopine synthase gene (OCS) of the Agrobacterium tumefaciens T-DNA | 1171 | 1191 | (+) | 0.743 | tgacgaaactttcAACGtgca |
| OPAQ | Opaque-2 like transcriptional activators | 1179 | 1195 | (+) | 0.833 | ctttcaACGTgcatgat |
| PSRE | Pollen-specific regulatory elements | 917 | 933 | (-) | 0.862 | atagaGAAAaattttat |
| ROOT | Root hair-specific cis-elements in angiosperms | 784 | 808 | (-) | 0.789 | aaatttattctcaatCACGtacatt |
| SBPD | SBP-domain proteins | 412 | 428 | (+) | 0.905 | agttaGTACaaaatatg |
| SEF4 | Soybean embryo factor 4 | 840 | 850 | (-) | 0.981 | aaTTTTtaata |
| SEF4 | Soybean embryo factor 4 | 1228 | 1238 | (-) | 0.992 | aaTTTTtatta |
| SPF1 | Sweet potato DNA-binding factor with two WRKY-domains | 388 | 400 | (-) | 0.877 | atTACTgtcattt |
| SPF1 | Sweet potato DNA-binding factor with two WRKY-domains | 239 | 251 | (-) | 0.873 | tgTACTcttattc |
| STKM | Storekeeper motif | 717 | 731 | (-) | 0.853 | aaaAAAAtaatttca |
| SUCB | Sucrose box | 781 | 799 | (-) | 0.815 | ctCAATcacgtacattaaa |
| SUCB | Sucrose box | 463 | 481 | (-) | 0.836 | atAATTcatattattcaat |
| SUCB | Sucrose box | 755 | 773 | (-) | 0.895 | aaAAATcacgtttaagatc |
| TEFB | TEF-box | 122 | 142 | (+) | 0.773 | gtATGGacaaaatagctatga |
| TELO | Telo box (plant interstitial telomere motifs) | 544 | 558 | (-) | 0.963 | ataaACCCtaggctt |
| TGACG | Uncommon JA-responsive motif | 139 | 134 | (+) | 1.0 | AATTGACGAAA |
| URNA | Upstream sequence element of U-snRNA genes | 188 | 204 | (+) | 0.76 | actatcCCAGatcctgc |
| WBXF | W Box family | 682 | 698 | (-) | 0.987 | attaaTTGAccagcgct |
| WBXF | W Box family | 1165 | 1181 | (+) | 0.96 | attaaTTGAcgaaactt |
| WBXF | W Box family | 1103 | 1119 | (-) | 0.97 | cttctTTGAcctattta |
| WNAC | Wheat NAC-domain transcription factors | 157 | 179 | (-) | 0.711 | tccgataattaTACGttaattaa |
