# Supplementary material for: The novel and taxonomically restricted Ah24 gene from grain amaranth (Amaranthus hypochondriacus) has a dual role in development and defense
Source: Front Plant Sci. 2015 Aug 5;6:602. doi: 10.3389/fpls.2015.00602 (PMC4524895; doi:10.3389/fpls.2015.00602)
Supplement: Supplementary file 13 [file Table2.DOCX]

**Table S2.** Most abundant cis-regulatory elements present in the promoter region of the *Ah24* gene.

| Family | Number of instances | Function |
| --- | --- | --- |
|  |  | **Jasmonic acid responses** |
| GBOX (G and C boxes, recognized by bZIP transcription factors [TFs]) | **1** | Essential for the regulation on jasmonic acid responses (JA). They have been identified in wound response defense genes such as those coding for protease inhibitors, in potato, leucine aminopeptidase, in tomato, and putrescine N-methyltransferase, in tobacco (^1^Memelink, 2009; Jakoby et al. 2002). |
| TGACG (uncommon JA-responsive motif) | **1** | Found to be essential for the induction of the *Nopaline synthase* and *Lipoxygenase 1* genes in tobacco, and barley, respectively (Wasternack and Parthier, 1997; Narusaka et al. 2004; Memelink, 2009). |
|  |  | **Regulation of development processes** |
| AHBP (Recognized by homeobox proteins in Arabidopsis) | **22** | Regulation of development processes (Palena et al., 1999; Trindade et al. 2003); present in nitric oxide-responsive genes many of which are involved in growth, development, and response to environmental stresses (Shi et al., 2012; Palmieri et al. 2008). |
| CCAF (Recognized circadian rhythm regulators) | **15** | Involved in the transition from vegetative to reproductive development. In general, regulated by light and Rubisco (Harmer and Kay 2005; McClung et al. 2006). |
| GT-Box (Identified in light-regulated  promoters) | **16** | Involved in plant growth regulation and response to various stress conditions. Found in the chalcone synthase gene in common and soya bean (Lawton et al. 1991**;** Singh 1998; Terzaghi and Cashmore 1995). |
| L1- Box (speficic motifs for the expression of L1 proteins) | **17** | L1 proteins regulate leaf and flower development processes. Present in Proteoderma factor 1, a proline-rich protein, which is exclusively found in the L1 layer of the apical meristem apical, the proteoderm and organ primordia (Abe et al. 2001; Lau et al. 2012). |
| MADS (Recognized by MADS-box TFs) | **13** | MADS-box genes encode a family of transcription factors which control diverse developmental processes in flowering plants ranging from root to flower and fruit development. Regulation of the transition from vegetative to reproductive development and generation of flowering structures and floral meristems (e.g. via Sepallata 3 TF) (Becker and Theißen 2003; Angenent et al. 2009; Gramzow and Theißen 2010, Smaczniak et al. 2012). |

**^1^Abe** et al. (2001) Plant J 26: 487-494; **Angenent** et al. (2009) PLoS Biol 7: 854-875; **Becker and Theißen** (2003) Mol Phylogenet Evol 29: 464-489; **Gramzow and Theissen** (2010) Genome Biol 11: 214; **Harmer and Kay** (2005) Plant Cell 17: 1926-1940; **Jakoby** et al. (2002) Trends Plant Sci 7: 106-111; **Lau** et al. (2012) Annu Rev Plant Biol 63: 483-506; **Lawton** et al. (1991) Plant Mol Biol 16: 235-249**; McClung** (2006) Plant Cell 18: 792-803; **Memelink** (2009) Phytochemistry 70: 1560-1570; **Narusaka** et al. (2004) Plant Mol Biol 55: 327-342; **Palena** et al. (1999) Biochem J 341: 81-87**; Palmieri** et al. (2008) J Exp Bot 59: 177-186; **Singh** (1998) Plant Physiol 118: 1111-1120; **Shi** et al. (2012) Plant Signal Behav 7: 438-440; **Terzaghi and Cashmore** (1995) Ann Rev Plant Physiol Plant Mol Biol 46: 445-474; **Trindade** et al. (2003) Gene 303: 77-87; **Smaczniak** et al. (2012) Development 139: 3081-3098; **Wasternack and Parthier** (1997) Trends Plant Sci 2: 302-307.
