# Supplementary material for: The novel and taxonomically restricted Ah24 gene from grain amaranth (Amaranthus hypochondriacus) has a dual role in development and defense
Source: Front Plant Sci. 2015 Aug 5;6:602. doi: 10.3389/fpls.2015.00602 (PMC4524895; doi:10.3389/fpls.2015.00602)
Supplement: Supplementary file 15 [file Table4.DOCX]

**Table S4.** Most up- and down-regulated genes identified in a microarray analysis of a highly *Ah24* overexpressing Arabidopsis transgenic plant (L15)

| Up-regulated genes | Z-score | Gene function | Reference(s) |
| --- | --- | --- | --- |
| ^2^1. Transposable element | 4.9 | Unknown |  |
| 2. MIR160/ MIR160A | 4.6 | Encodes a microRNA that targets several ARF family members (ARF10, ARF16, ARF17); regulates auxin signaling in the context of JA and ABA responses during plant development; Reprograms leaf growth during drought stress | Bertolini et al. 2013; Curaba et al. 2014 |
| 3. Imidazoleglycerol-phosphate dehydratase | 4.45 | Histidine biosynthesis | Rajani et al. 2013 |
| 4. *Sleepy 1* (*SLY1*) | 4.44 | Encodes a F-box family protein that act as positive regulators of gibberellin signaling | Ariizumi et al. 2011 |
| 5. *RABA1i* | 4.33 | Rab GTPases are important determinants of membrane identity and membrane targeting; their activation is essential for development in Arabidosis. | Goh et al 2007; Woollard and Moore 2008; Cui et al. 2014 |
| 6. Cytochrome P450, family 714, subfamily A, polypeptide 2 | 4.20 | Encodes a gibberellin 13-oxidase that regulates gibberellin activity | Zhang et al. 2011b; Magome et al. 2013 |
| 7. Nucleotide-sugar transporter family protein | 4.03 | Probable sugar phosphate/ phosphate translocator. Believed to be involved in cell wall (cellulose) biosynthesis and to have a general role in development of eukaryotes. | Liu et al. 2010; Zhang et al. 2011a |
| 8. *Cytokinin response factor 2* | 3.96 | Constitute AP2/ERF ethylene-responsive transcription factor genes that regulate leaf development as part of the cytokinin signal transduction pathway in Arabidopsis; involved in biotic and abiotic stress responses. | Rashotte and Goertzen 2010; Cutcliffe et al. 2011; O’Brien and Benková 2013 |
| 9. *Kinesin 5* | 3.87 | Proposed to be needed for cellulose microfibril deposition; found to be involved in mitosis in plants; in Arabidopsis, they contribute directly or indirectly to cell division and cell growth in various tissues; predicted to regulate cell expansion in cucumber fruits. | Zhong et al. 2002; Lee and Liu 2004; Bannigan et al. 2007; Yang et al. 2013 |
| 10. *SEUSS-like 1* (*SLK1*) | 3.74 | Part of transcriptional adaptor plant proteins proposed to function in diverse developmental processes and in abiotic stress responses | Bao et al. 2010; Shrestha et al. 2014 |
| 11. Coding for C2H2 and C2HC zinc fingers superfamily protein | 3.73 | Constitute one of the largest families of transcriptional regulators in plants. Many of these proteins function as part of a large network that senses and responds to different environmental and phytohormone stimuli to regulate development and growth. | Ciftci-Yilmaz and Mittler 2008; Li et al. 2013a |
| 14. Transposable element | 3.68 | Unknown |  |
| Down-regulated genes | **Z-score** | **Gene function** | **Reference(s)^1^** |
| 1. RING-H2 finger A1A | -6.24 | The RING (Really Interesting New Gene) finger proteins have a C3HC4 zinc finger and act as E3-ubiquitin ligases. Some are essential for seed development in Arabidopsis; identified as possible QTL for grain yield under drought in rice; a RING Zinc Finger Protein 1 from chilli pepper enhanced growth when overexpressed in tobacco | Zeba et al. 2009; Agarwal et al. 2011; Swamy et al. 2011 |
| 2. MIR398B | -5.66 | A miRNA proposed to regulate plant responses to oxidative, water deficit, salt and abscisic acid stresses, ultraviolet, copper and phosphate deficiency, high sucrose and bacterial infection. Found to have a diurnal oscillation *in A. thaliana*. | Siré et al. 2009; Zhu et al. 2011 |
| 3. TLD-domain containing nucleolar protein | -5.36 | Identified as part of a battery of microRNAs involved in cold, wounding and salt stresses in wheat. | Wang et al. 2014 |
| 4. MIR164/MIR164B | -5.15 | Induced in response stress and to insect attack and bacterial infection in Arabidopsis thaliana | Liu et al. 2008; Barah et al. 2013 |
| 5. AT hook motif DNA-binding family protein (*AGF1*) | -5.11 | In plants, AT-hook proteins plays a role in developmental processes, such as flowering transition and stress responses. | Yun et al. 2012 |
| 7. Zinc finger (AN1-like) family protein | -5.00 | Zinc-finger (AN1-like)-like proteins are known to be involved in stress tolerance. | Swamy et al. 2011 |
| 7. Disease resistance protein (TIR-NBS-LRR class) family | -4.67 | Believed to have a dual role in development and pathogen immunity  in Arabidopsis | Develey-Rivière and Galiana 2007; Xin and He 2013 |
| 8. crooked neck protein, putative / cell cycle protein, putative | -4.59 | Regulation of progression through the cell cycle. Identified in water deficit-stressed sugarcane leaves at tillering stage and in herbicide-treated rice root tips; also, involved in the photoperiodic control of floral initiation in *Pharbitis nil*. | Higuchi et al. 2007; Kojima et al. 2009; Prabu et al. 2011 |
| 12. WD40/YVTN repeat-like-containing domain; Bromodomain | -4.18 | Identified in in the S. lycopersicum stem trichome transcriptome; probably involved in terpene biosynthesis and as a marker of development in a rattan species. Induced in chrysanthemums infected by the Chrysanthemum stunt viroid. | Li et al. 2013b; Jo et al. 2014; Spyropoulou et al. 2014 |
| 13. KH domain-containing protein | -3.98 | KH domain-containing RNA binding proteins were identified as flowering regulators in Arabidopsis; also involved in vegetative and reproductive development and in plant-environment interactions. | Lorković 2009; Ambrosone et al. 2012 |
| 14. C2H2-like zinc finger protein | -3.94 | See above |  |
| 15. BTB/POZ domain-containing protein | -3.89 | Broad complex/Pox virus and Zinc finger (BTB/POZ) domain. Involved in protein-protein interactions. Some are putative substrate adaptors for cullin 3 ubiquitin ligases, known to control aspects of plant development via partly by the control of phytohormone signal transduction pathways. | Schwechheimer and Calderón Villalobos 2004; Figueroa et al. 2005; Thomann et al. 2005 |
| 17. Cysteine proteinases superfamily protein | -3.77 | Biochemical evidence suggests a role for cysteine proteases in plant programmed cell death (PCD). PCD is activated during the hypersensitive response to pathogen attack, tracheary-element differentiation, and senescence. Also found to regulate ethylene signaling. | Estelle 2001; Matarasso et al. 2005 |
| 18. Syntaxin/t-SNARE family protein | -3.70 | Plants contain a large set of SNAREs thought to be involved in membrane trafficking in plants mostly associated with different vacuole types. Also, involved in membrane fusion guidance molecule in ABA regulation of ion channels. Believed to be required for auxin-dependent developmental processes, gravitopism and pathogen defense. | Luan 2002; Bassham and Blatt 2008; Leshem et al. 2010 |
| 19. Pentatricopeptide repeat (PPR) superfamily protein | -3.70 | Abundant RNA-binding proteins that have a range of essential functions in post-transcriptional processes (including RNA editing, RNA splicing, RNA cleavage and translation). Required for seed and chloroplast development. | Schmitz-Linneweber and Small 2008 |
| 20. Flavin-binding monooxygenase family protein | -3.63 | Respiratory protein found to be up-regulated by drought, cold or high-salinity stress in Arabidopsis. May regulate plant growth and development in Arabidopsis, via the YUCCA Flavin-containing monooxygenase is involved in auxin biosynthesis. | Seki et al. 2002; Dai et al. 2013 |
| 22. *ZIM-LIKE 2* or *TIFY* | -3.55 | Available data suggest a regulatory function for the members of this family. Experimental data suggest that TIFY proteins might be involved in various developmental processes and may play a role in jasmonic acid signaling. | Vanholme et al. 2007 |

**^1^Agarwal** et al. (2011) Bioessays 33: 189-202; **Ambrosone** et al. (2012) Plant Sci 182: 12-18; **Ariizumi** et al. (2011) Plant Physiol 155: 765-775; **Bannigan** et al. (2007) J Cell Sci 120: 2819-2827; **Bao** et al. (2010) Plant Physiol 152: 821-836; **Barah** et al. (2013) PLoS ONE 8: e58987; **Bassham and Blatt** (2008) Plant Physiol 147: 1504-1515; **Bertolini** et al. (2013) Mol Plant 6: 423-443; **Ciftci-Yilmaz and Mittler** (2008) Cell Mol Life Sci 65: 1150-1160; **Cui** et al. (2014) Plant Cell 26: 2080-2097; **Curaba** et al. (2014) J Exp Bot 65: 1425-1438; **Cutcliffe** et al. (2011) J Exp Bot 62: 4995-5002; **Dai** et al. (2013) J Biol Chem 288: 1448-1457; **Develey-Rivière and Galiana** (2007) New Phytol 175: 405-416; **Estelle** (2001) Curr Opin Plant Biol 4: 254-260; **Figueroa** et al. (2005) Plant Cell 17: 1180-1195; **Goh** et al. (2007) Plant Cell 19: 3504-3515; **Higuchi** et al. (2007) Plant Biotechnol 24: 201-207; **Jin** et al. (2009) Plant Cell 21: 2072-2089; **Jo** et al. (2014) Plant Omics 7: 1-11; **Kojima** et al. (2009) Pesticide Biochem Physiol 93: 58-64; **Lee and Liu** (2004) Plant Physiol 136: 3877-3883; **Leshem** et al. (2010) J Exp Bot 61: 2615-2622; **Li** et al. (2013a) Plant Omics J 6: 474-480; **Li** et al. (2013b) Mol Breeding 31: 867-877; **Liu** et al. (2008) RNA 14: 836-843; **Liu** et al. (2010) Semin Cell Dev Biol 21: 600-608; **Lorković** (2009) Trends Plant Sci 14: 229-236; **Luan** (2002) Plant Cell Environ 25: 229-237; **Magome** et al. (2013) Proc Natl Acad Sci USA 110: 1947-1952; **Matarasso** et al. (2005) Plant Cell 17: 1205-1216; **O’Brien and Benková** (2013) Front Plant Sci 4: 451; **Prabu** et al. (2011) Plant Mol Rep 29: 291-304; **Rajani** et al. (2013) Recent Res Devel Plant Physiol 5: 51-62; **Rashotte and Goertzen** (2010) BMC Plant Biol 10: 74; **Schmitz-Linneweber and Small** (2008) Trends Plant Sci 13: 663-670; **Schwechheimer and Calderón Villalobos** (2004) Curr Opin Plant Biol 7: 677-686; **Seki** et al. (2002) Plant J 31: 279-292; **Shrestha** et al. (2014) BMC Plant Biol 14: 54; **Siré** et al. (2009) FEBS Lett 583: 1039-1044; **Spyropoulou** et al. (2014) BMC Genomics 15: 402; **Swamy** et al. (2011) BMC Genomics 12: 319; **Thomann** et al. (2005) FEBS Lett 579: 3239-3245; **Vanholme** et al. (2007) Trends Plant Sci 12: 239-244; **Wang** et al. (2014) Plant Physiol Biochem 80: 90-96; **Woollard and Moore** (2008) Curr Opin Plant Biol 11: 610-619; **Xin and He** (2013) Annu Rev Phytopathol 51: 473-498; **Yang** et al. (2013) J Exp Bot 64: 4541-4557; **Yun** et al. (2012) J Biol Chem 287: 15307-15316; **Zeba** et al. (2009) Planta 229: 861-871; **Zhang** et al. (2011a) Proc Natl Acad Sci USA 108: 5110-5115; **Zhang** et al. (2011b) Plant J 67: 342-353; **Zhong** et al. (2002) Plant Cell 14: 3101-3117; **Zhu** et al. (2011) Physiol Plant 143: 1-9.

^2^Numbering of the genes is not consecutive. It represents their position in the microarray according to level of induction or repression.
