# Supplementary material for: The novel and taxonomically restricted Ah24 gene from grain amaranth (Amaranthus hypochondriacus) has a dual role in development and defense
Source: Front Plant Sci. 2015 Aug 5;6:602. doi: 10.3389/fpls.2015.00602 (PMC4524895; doi:10.3389/fpls.2015.00602)
Supplement: Supplementary file 16 [file Table5.DOCX]

| **Table S5.** Sequence of the primers employed for PCR amplification in this study. The table includes oligonucleotide sequences employed for diverse purposes, including qRT-PCR, RACE, Genome Walking and confirmation of heterologous expression in Arabidopsis and tobacco. | | | |
| --- | --- | --- | --- |
| **Primer**  **nomenclature** | **5’-3’ oligonucleotide sequence** | **Primer**  **nomenclature** | **5’-3’ oligonucleotide sequence** |
| **Expression levels in *Ah* by qRT-PCR** | | **Complete *Ah24* cDNA sequence (RACE) in *Ah*** | |
| qAh24-F | GATTTAATTGAGATGGCTGAAA | Race5’Ah24 | GGACCATCAGCATAAGCAACACCCCA |
| qAh24-R | GTTAGTGCAGCTTGTTCGC | Race3’Ah24 | ACATTTTTGGGCAGGTCGGGCAGTC |
| qACT-F | CGTGACCTGACTGATTACCTTA |  | |
| qACT-R | GCCATTGAGAAGAACTACGAGC |  |  |
| qTUB-F | TCTCAGCAGTATGTCTCCCTCA |  |  |
| qTUB-R | AAGATGAGCACCAAAGAAGTAGA |  |  |
| **Cloning of *Ah24* in tobacco and Arabidopsis** | | **Complete *Ah24* gene (Genome walking) in *Ah*** | |
| Ah24pMDC-F | ATTCAGCAAAAAGGATCT | GWP1Ah24-R | GTTTGTTGGCATTTTACTTCTTTGACCT |
| Ah24pMDC-R | GCAAACACACTTTATATTCC | GWP2Ah24-R | TTAGCTGAATCATGCACGTTGAAAGTTTCG |
| Ah24pB7-F | ATTCAGCAAAAAGGATCT | GW1Ah24-R | TTTCAGCCATCTCAATTAAATCTTTTTTGC |
| Ah24pB7-R | GGAATATAAAGTGTGTTTGC | GW2Ah24-R | AGCATTCTTATCCTCCACACTGTACACTTG |
|  | | GW3Ah24-R | CGACCTGCCCAAAAATGTTCTTTCTC |
|  |  | GW4Ah24-R | GTGCAATGGCGGACAGGACGGTGACT |
|  |  | GW5Ah24-R | GATCAACGGAAAGTAGCCCAAGCATTG |
|  |  | GW6Ah24-R | TCAATCTCAGCAGTACATTTTCCACCAAG |
|  |  | GW1Ah24-F | CTTGGTGGAAAATGTACTGCTGAGATTGA |
|  |  | GW2Ah24-F | CAATGCTTGGGCTACTTTCCGTTGATC |
|  |  | GW3Ah24-F | GAGAAAGAACATTTTTGGGCAGGTCG |
|  |  | AP2adaptador | ACTATAGGGCACGCGTGGTCGACGGCCCGGGCTGGT |
|  |  | GW4Ah24-F | AGTCACCGTCCTGTCCGCCATTGCAC |
| **Expression levels by qRT-PCR in Arabidopsis and tobacco(including microarray validation)** | | **Expression levels by qRT-PCR in Arabidopsis and tobacco(including microarray validation)** | |
| AtMIR160a-F  AtMIR160a-R  AtIPD-F  AtIPD-R  AtSLY-F  AtSLY-R  AtP450-F  AtP450-R  AtCRF2-F  AtCRF2-R  AtSLK1-F  AtSLK1-R  AtRAB1i-F  AtRAB1i-R  AtNSTF-F  AtNSTF-R  AtKin5-F  AtKin5-R  AtRhoGAP-F  AtRhoGAP-R  AtDEADbox-F  AtDEADbox-R  AtRHA1A-F | ATTCCTCCACAAGAGGGAGA  GAGGAATAGAAACAATCTATGGCA  CAGCTTCGCCAATCTATCTCT  CCAGTACCATCCAAATTAATCTTC  ATGAAGCGCAGTACTACCGA  CACCTCGTACACTAGATTCTCGTC  GTTAAAATGGAGAGTTTGGTTGTT  TTCACGCCTTGCAACTTTAA  ATGGAAGCGGAGAAGAAAAT  TTCTTCTTCTTCGTCGTCACT  GTTATCTGCTCTCGTGGGTG  TATTGATGCCACTGCTCCTCT  GAGCAGAAGACGATTATGATTACC  TTTGAGCCTTGACGATCTTAT  TAAGCGAAGGCGTGATTAAGA  GAGCAAAAGGACATGTGGATC  CCTAAGGTGAAGCAATCCATC  GTCTCCTTCCTTCGCTTGTC  TGTGTACTTCTGCAATGGAGG  CTTCTTCCAGGATGTCCAAC  CAGTGAAATCAGGAATTTGGTATG  GAAGCCGGTCTCTCGTACTG  CTCTAAATCATGGGTCTTCCTG | AtRHA1A-R  AtmiR398b/c-F  AtmiR398b/c-R  AtCitOxi-F  AtCitOxi-R  AtTLD-F  AtTLD-R  AtAThook-F  AtAThook-R  AtZnAN1-F  AtZnAN1-R  AtAMP-F  AtAMP-R  AtC_2_H_2_-F  AtC_2_H_2_-R  AtSNARE-F  AtSNARE-R  NtL25-F  NtL25-R  NtEF1α-F  NtEF1α-F  Hyg-F  Hyg-R | CTAAAGAAACCGATGACGTAAAGT  GGATCTCGACAGGGTTGATATG AAGAGCTCAGCAGGGGTGACCTG AACAATCTCACTCGAAATTCCTT  ATAACGAAAGAAGATGCGGAA  GGGTTGAAGTTGTAATTTTGAAG  AGCAGAAATGAAGCGATGAT  TAGGGTCCCCATCGAATTT  GAGTTTGAGGAGGAGGAGGA  CTTATGGCAGGAGGAGGAA  TGTGACTTGTATGATCTATGCTCC  GAGGCTTTCGATACAACCAA  GCTCACGTTTGCTGTAGAATATC  TTCCCAGCTATGTCCTTCAC  ACTTCTGCATCTGGATCTGG  TGATGACGACGAGTTTGGAT  GTGAAGATCTCTATGAAGCTGCTC  CCCTCACCACAGAGTCTGC  AAGGGTGTTGTTGTCCTCAATC  TGAGATGCACCACGAAGCT  CAACATTGTCACCAGGAAGTG  GCGAAGAATCTCGTGCTTT  TCGCTAAACTCCCCAATGTC |
| **Cloning of Ah24-GFP fusion in Arabidopsis** | |  | |
| Ah24GFP-F  Ah24GFP-R | ATGGCTGAAATTGAAGCACAAG  TCATCGGAAAGTAGCCCAAG |  |  |
